# Melatonin attenuates diabetic cardiomyopathy by increasing autophagy of cardiomyocytes via regulation of VEGF-B/GRP78/PERK signaling pathway

**DOI:** 10.1186/s12933-023-02078-x

**Published:** 2024-01-09

**Authors:** Shengzheng Zhang, Wencong Tian, Xianxian Duan, Qian Zhang, Lei Cao, Chunlei Liu, Guangru Li, Ziwei Wang, Junwei Zhang, Jing Li, Liang Yang, Yang Gao, Yang Xu, Jie Liu, Jie Yan, Jianlin Cui, Lifeng Feng, Chang Liu, Yanna Shen, Zhi Qi

**Affiliations:** 1 Department of Molecular Pharmacology, School of Medicine, Nankai University, Tianjin, 300071 China; 2 Tianjin Key Laboratory of General Surgery in Construction, Tianjin Union Medical Center, Tianjin, 300000 China; 3 School of Medical Technology, Tianjin Medical University, Tianjin, 300203 China; 4 Key Laboratory of Emergency and Trauma of Ministry of Education, Hainan Medical University, Haikou, 571199 China; 5 Xinjiang Production and Construction Corps Hospital, Xinjiang, 830092 China

**Keywords:** Melatonin, Diabetic cardiomyopathy, VEGF-B, Autophagy, Endoplasmic reticulum stress

## Abstract

**Aims:**

Diabetic cardiomyopathy (DCM) is a major cause of mortality in patients with diabetes, and the potential strategies for treating DCM are insufficient. Melatonin (Mel) has been shown to attenuate DCM, however, the underlying mechanism remains unclear. The role of vascular endothelial growth factor-B (VEGF-B) in DCM is little known. In present study, we aimed to investigate whether Mel alleviated DCM via regulation of VEGF-B and explored its underlying mechanisms.

**Methods and results:**

We found that Mel significantly alleviated cardiac dysfunction and improved autophagy of cardiomyocytes in type 1 diabetes mellitus (T1DM) induced cardiomyopathy mice. VEGF-B was highly expressed in DCM mice in comparison with normal mice, and its expression was markedly reduced after Mel treatment. Mel treatment diminished the interaction of VEGF-B and Glucose-regulated protein 78 (GRP78) and reduced the interaction of GRP78 and protein kinase RNA -like ER kinase (PERK). Furthermore, Mel increased phosphorylation of PERK and eIF2α, then up-regulated the expression of ATF4. VEGF-B^−/−^ mice imitated the effect of Mel on wild type diabetic mice. Interestingly, injection with Recombinant adeno-associated virus serotype 9 (AAV9)-VEGF-B or administration of GSK2656157 (GSK), an inhibitor of phosphorylated PERK abolished the protective effect of Mel on DCM. Furthermore, rapamycin, an autophagy agonist displayed similar effect with Mel treatment; while 3-Methyladenine (3-MA), an autophagy inhibitor neutralized the effect of Mel on high glucose-treated neonatal rat ventricular myocytes.

**Conclusions:**

These results demonstrated that Mel attenuated DCM via increasing autophagy of cardiomyocytes, and this cardio-protective effect of Mel was dependent on VEGF-B/GRP78/PERK signaling pathway.

**Supplementary Information:**

The online version contains supplementary material available at 10.1186/s12933-023-02078-x.

## Introduction

Diabetic cardiomyopathy (DCM), characterized by abnormal myocardial structure and function, is one of the major complications of diabetes mellitus (DM) that can result in heart failure [1]. Multiple factors, such as cardiac insulin resistance, mitochondrial dysfunction, and oxidative stress, are responsible for the pathogenesis of DCM [2]. However, a single treatment strategy of strict glycemic control does not significantly reduce the risk of cardiovascular events [3], and the therapeutic effect for DCM has not significantly improved in the last few decades. Therefore, seeking new strategies and novel therapeutic targets for DCM are crucial in the clinical treatment.

Melatonin (Mel) is mainly synthesized by the pineal gland. It functionally serves as free radical scavenger, anti-inflammatory agent and immunoregulating molecule [4]. Mel has primarily been explored for its involvement in sleep and circadian rhythm regulation, encompassing both acute effects and circadian phase-shifting effects [5]. Mel also has therapeutic effects on type 2 diabetes [6], ischemia–reperfusion injury [7] and heart failure [8]. It has been proved that Mel administration could alleviate DCM-induced cardiac dysfunction [9]. However, the underlying mechanism of Mel on DCM needs further clarification.

Vascular endothelial growth factor-B (VEGF-B) is a member of the VEGF family and mainly expressed in heart, skeletal muscle, brown adipose tissue, pancreas and prostate [10, 11]. VEGF-B can bind to the transmembrane protein tyrosine kinase receptor VEGFR1 and further activate several downstream signaling pathways, such as p38/MAPK, ERK/MAPK, PKB/AKT and PI3K [12]. Researchers' understanding for VEGF-B function has evolved from an inert gene to a novel therapeutic target for myocardial infarction [13]. Two consecutive articles have shown that VEGF-B can regulate fatty acid transport in endothelial cells [14], and inhibition of VEGF-B can improve muscle insulin sensitivity [15]. Therefore, VEGF-B may serve as a potential target for type 2 diabetes. However, few studies focused on the role of VEGF-B in DCM. Importantly, the relationship between Mel treatment and VEGF-B signaling pathway has not been reported yet.

More recently, the disorder of endoplasmic reticulum (ER) stress and unbalanced autophagy were considered to contribute to the onset and progression of DCM [16, 17]. One form of ER stress is known as protein kinase RNA-like ER kinase (PERK)/translation initiation factor eIF2α pathway, which has a role in diabetes and diabetic complications [18]. Under ER stress, glucose regulated protein 78 (GRP78) in the ER was dissociated from PERK and resulted in phosphorylation of PERK, thereafter the PERK/eIF2α pathway was initiated to halt mRNA translation by preventing 80 s ribosome assembly but paradoxically increased ATF4 expression [19, 20]. ATF4 is a member of the cAMP-responsive element-binding protein family of basic zipper-containing proteins, and it regulates a variety of genes involved in various physiological processes, including apoptosis, lipid metabolism, and obesity [21–23]. The transcription factor ATF4 further transactivates unfolded protein response (UPR) target genes involved in autophagy [24]. Interestingly, ATF4-induced autophagy was a critical regulatory factor for cardiomyocyte size in the stressed heart [25].

Autophagy, as a protective mechanism for cellular metabolism and the renewal of organelles, is tightly regulated by many positive and negative regulators including ATG protein family, beclin1 and LC3-II. They were involved in the initiation, vesicle formation and elongation, nucleation, autophagosome formation, and autophagosome-lysosome fusion [26]. The UPR inhibits cellular protein synthesis and assists degradation of misfolded or damaged proteins, ultimately increases cell apoptosis and autophagy [27]. It has been reported that sustaining constitutive basal autophagy were beneficial for alleviation of DCM [28]. The activation of the autophagic response serves as a compensatory feedback mechanism to protect the cell from undergoing apoptosis [29]. Nevertheless, the relationship between VEGF-B and ER stress-induced autophagy in DCM remains unknown.

In this study, we explored whether Mel had cardioprotective effect on DCM. Importantly, we investigated the crucial role of VEGF-B in the protective effect of Mel against DCM and explored the relationship between VEGF-B/GRP78/PERK axis and autophagy of cardiomyocytes in DCM.

## Materials and methods

### Animals

This study was approved by Nankai University Ethics Committee on Animal Care (Permit number: 10011), and all animals received humane care in adherence with the guidelines for laboratory animals in Nankai University. Investigators designed and performed experiments strictly in compliance with the National Institutes of Health Guide for the Care and Use of Laboratory Animals. All mice were housed under a controlled temperature (25 °C) in a 12 h light/dark cycle. Eight-week C57BL/6 wild type (WT) mice, weighing 20 g were purchased from SPF Biotechnology (Beijing, China). All mice were randomly divided into the following groups (n = 6 each): control group (Con), diabetic mice group (DM), diabetic mice with Mel treatment group (DM + Mel), diabetic VEGF-B knock out mice group (DM + VEGF-B^−/−^), diabetic mice with Mel and recombinant adeno-associated virus serotype 9 (AAV9)-VEGF-B overexpression treatment group (DM + Mel + AAV-VEGF-B), diabetic mice with Mel and GSK2656157 (GSK) treatment group (DM + Mel + GSK), diabetic VEGF-B knock out mice with GSK treatment group (DM + VEGF-B^−/−^ + GSK).

### Genetically modified mice

VEGF-B global knockout mice were purchased from Shanghai Model Organisms Center Inc. (Shanghai, China) (C57BL/6 background). PCR was used to screen VEGF-B^−/−^ mice. Age-matched male mice (n = 6) were used in all animal experiments. Genotyping of WT, VEGF-B^±^ and VEGF-B^−/−^ was shown in Additional file 2: Table S1.

### Experimental T1DM mice model

T1DM mice model was constructed by intraperitoneal injection of streptozotocin (STZ, 150 mg/kg, formulated in 0.1 M citrate buffer (PH = 4.5)). Mice with fasting blood glucose ≥ 11.1 mmol/L for 3 consecutive days were considered to be T1DM mice [30]. Melatonin was initially dissolved in DMSO and then diluted in saline to a final concentration of 2 mg/ml. After model establishment (one week after STZ injection), mice in DM + Mel group were orally administrated with Mel (20 mg/kg) daily at 10:00 am for 7 weeks. Other DM mice were treated with saline in the same volume (0.1 ml per 10 g body weight). Fasting blood glucose, non-fasting blood glucose and body weight were recorded every week. Mice were anaesthetized using 3% inhaled isoflurane combined with 100% oxygen. Upon attaining an appropriate plane of anesthesia, the heart was removed. Blood samples were collected for measurement of creatine kinase (CK-MB) levels. Part of the heart tissues were fixed in 4% paraformaldehyde for histopathology and immunohistochemistry test. Parts of the heart tissues were snap-frozen for later western blot and real-time PCR analysis.

### AAV9-VEGF-B construction and viral delivery

AAV9 vectors were purchased from Hanbio Biotechnology Co., China. WT mice were injected with 0.1 ml of AAV9- cTnT-GFP-Flag-VEGF-B or GFP empty vector through the tail vein at a dose of 2 × 10^12^ vg/kg, in the third week after T1DM model established.

### Cardiac function detection

Mice were anesthetized using 3% isoflurane before assessing cardiac function via transthoracic echocardiography at the 8weeks. (vevo2100, Visualsonics, USA). Images were obtained from left ventricular parasternal long axis M-mode levels. A minimum of five consecutive cardiac cycles were measured for ejection fraction (EF) and fractional shortening (FS). Other echocardiographic parameters in mice were shown in Additional file 3: Table S2.

### Detection of CK-MB in serum

The concentrations of CK-MB in serum were detected by commercial ELISA kits (ELK Biotechnology, Wuhan, China) according to the manufacturers’ instructions.

### Isolation and culture of NRVMs

Neonatal rat ventricular myocytes (NRVMs) were obtained from neonatal rats within 24 h (SPF Biotechnology, Beijing, China) as described previously [31]. Cells were cultured in Dulbecco’s Modified Essential Medium (DMEM) with 5.5 mM D-glucose, supplemented with 1% penicillin–streptomycin (PS) and 1% insulin-Transferrin-Selenium (ITS) in a humidified atmosphere of 5% CO_2_ at 37 °C. Cells were starved for 12 h without ITS, and randomized into different experimental groups: Glucose were added to 33 mM for 4h after Mel treatment (100 µM) for 4 h or not. Rapamycin (250 nM) was treated for 24 h, 3-Methyladenine (3-MA) (5 nM) was treated for 3 h, GSK (100 nM) was treated for 12 h. Mel, 3-MA, Rapamycin, GSK were purchased from MedChemExpress (Monmouth Junction, USA).

### Quantitative real-time PCR

The protocol for quantitative real-time PCR has been described previously [32]. In brief, total RNA was isolated using Trizol reagent (Solarbio Biotechnology Co., Ltd, Beijing, China) from mice heart tissues and NRVMs. The mRNA (500 ng) was used for cDNA synthesis by reverse transcription system (Yeasen Biotech, China). Real-time polymerase chain reaction was performed using SYBR Green Master Mix (Yeasen Biotech, China) in a Roche LightCycler 96 detection system. The primers used for the detected genes were listed in Additional file 4: Table S3.

### Histological and immunohistochemistry assessments

The heart tissue was fixed with 4% formaldehyde, followed by dehydrated, transparent, and embedded with paraffin. Finally, the heart tissue was cut into 5 μm thick samples for subsequent experiments. For immunohistochemical staining, the tissue sections were baked at 65 °C for 2 h, deparaffinized and rehydrated, and then performed to antigen retrieval in 0.01 M citrate buffer (Solarbio Biotechnology Co., Ltd, Beijing, China). Then, tissues were blocked with 5% goat serum for 1 h and then incubated with VEGF-B antibody (1:200) at 4 °C overnight. Afterwards, the slices were stained with hematoxylin and eosin (H&E) (Beyotime Biotechnology, Shanghai, China), Masson staining (ZSGB-BIO, Beijing, China), WGA-FITC staining (Sigma-Aldrich, St. Louis, MO, USA), and Sirius red staining (Solarbio Biotechnology Co., Ltd, Beijing, China) according to the manufacturer’s instructions. In the histological staining described above, the magnifications for H&E, Masson, and Sirius Red were 200x, the magnification for WGA staining was 400x. The image was captured under microscope (Nikon, Tokyo, Japan).

### Small interfering RNA (siRNA) transfection

VEGF-B siRNA was purchased from GenePharma (Shanghai, China). Briefly, NRVMs were cultured in opti-MEM for 2 h before siRNA transfection. VEGF-B siRNA (50 nM) or negative control (NC) siRNA was mixed with RFect siRNA/miRNA Transfection Reagent (Baidai Biotechnology, Changzhou, China) according to manufacturer’s instructions. The cells were incubated with the transfection mixture for 6 h and then washed with DMEM medium. The cells were incubated for an additional 48 h before harvest.

### Western blot

NRVMs or homogenized heart tissues were lysed. Lysates were separated by 8–15% SDS-PAGE gels and then electro-transferred to nitrocellulose membranes, subsequently blocked with 5% non-fat milk-TBST buffer for 1 h at room temperature. The membranes were incubated with primary antibodies at 4 °C overnight. Then the membranes were incubated with horseradish peroxidase-conjugated secondary antibody (1:5000) for 1h at room temperature. The target bands were detected by the ECL solution system (Shandong Sparkjade Biotechnology Co., Ltd., Shandong, China) and analyzed using Image J. LC3 (2775s), P62 (39749s), PERK (3192s), p-PERK (3179s), eIF2α (5324s), p-eIF2α (3398s), ATF4 (11815s) were obtained from Cell Signaling Technology; VEGF-B (ab110649), GRP78 (ab21685) were purchased from Abcam; β-actin (sc47778), VEGF-B (sc-80442, 1:200 for IP) were purchased from Santa Cruz Biotechnology; Flag-tag (390002), HA-tag (301113) were obtained from ZENBIO; GST-tag (T0007), Flag-tag (T0053, 1:100 for IP) were purchased from Affinity; GRP78 (66574–1-Ig, 1:50 for IP) was purchased from Proteintech, HA-tag (AE008, 1:100 for IP) was purchased from Abclonal.

### Immunofluorescence and autophagy analysis

Paraformaldehyde-fixed, Triton X-100-permeabilized NRVMs were subjected to immunofluorescence staining to analyze the expression and localization of VEGF-B, GRP78 and ATF4. Hoechst 33342 (Yeasen Biotech, China) and 6-diamino-2-phenylindole (DAPI) were used to nuclear staining (St. Louis, MO, USA). Cyto-ID Autophagy Detection Kit (Enzo Life Sciences, NY, USA) was used to detect the autophagic flux, according to the manufacturer’s protocol. The confocal microscope (Olympus FV1000) was used for capturing images with high resolutions.

### Calcein-AM/PI double staining

NRVMs were incubated in PBS containing 2 μg/ml calcein AM (Sigma-Aldrich, St. Louis, MO, USA) at 37 °C for 30 min and 1 μg/ml propidiumiodide (PI) (Sigma-Aldrich, St. Louis, MO, USA) at 37 °C for 5 min. After washing with PBS, cells were examined by fluorescence microscopy. Five random fields were observed, PI positive area and the global cells area were measured by Image J software. PI positive area rate was defined as the percentage of the PI positive area to the whole cells area.

### Immunoprecipitation

We performed immunoprecipitation in NRVMs and 293T cells. 293T cells were transfected with Flag-VEGF-B or HA-GRP78 for 24 h. Cells were collected and then lysed in RIPA buffer supplemented with a complete protease inhibitor cocktail (beyotime, shanghai, China). After pre-clearing with protein A/G agarose beads (sc-2003, Santa) for 2  h at 4 °C, whole-cell lysates were used for immunoprecipitation with the indicated antibodies. Generally, commercial antibody (1 μg) was added to 500 μg of proteins and incubated at 4 °C for 12 h. After incubation with protein A/G agarose beads for 6 h at 4℃, agarose beads were extensively washed with lysis buffer and eluted with 2 × SDS loading buffer by boiling for 10 min. Western blot was performed using the precipitated proteins and cell lysates.

### GST pull down

Rat GRP78-full length (FL) (28–654), GRP78-NBD (28–405), GRP78-SBD (422–654) proteins were cloned into pGEX-6p-1 vector containing a GST-tag. All constructs were expressed in Transetta (DE3) cells (TransGen Biotech, Beijing, China), and were grown at 37 °C for 5 h, then 0.8 mM IPTG was added at 37 °C for 3 h followed by 20 ℃ for 24 h and then purified with glutathione Sepharose 4B beads (Solarbio biotechnology Co., Ltd, Beijing, China). For overexpression of Flag-VEGF-B in the 293T cells, cells were transfected with 20 μg of Flag-VEGF-B per plate. Transient transfection was performed using Lipofectamine 2000 (Thermo, USA) according to the manufacturer’s instructions. The cells were then collected at 30 h post transfection and lysed at 4 °C. Flag-VEGF-B protein was rotated with GST-GRP78-FL, GST-GRP78-NBD, GST-GRP78-SBD at 4 °C for 4 h. After centrifugation and three washes, the beads were eluted with 50 μl of 2 × SDS-PAGE loading buffer and then boiled for 10 min, followed by western blot.

### Statistical analysis

All the data were presented in box-plot. Data presented in the present study were representative of at least 3 independent experiments and expressed as mean ± standard deviation (SD). Data were tested for normality using the Shapiro–Wilk test. Statistical analysis of the data involved performing a one-way analysis of variance (ANOVA) followed by a Tukey post-hoc test for comparisons among multiple groups. Statistical significance was defined as a p-value < 0.05.

## Results

### Mel alleviated cardiac dysfunction in diabetic mice.

**Fig. 1:**
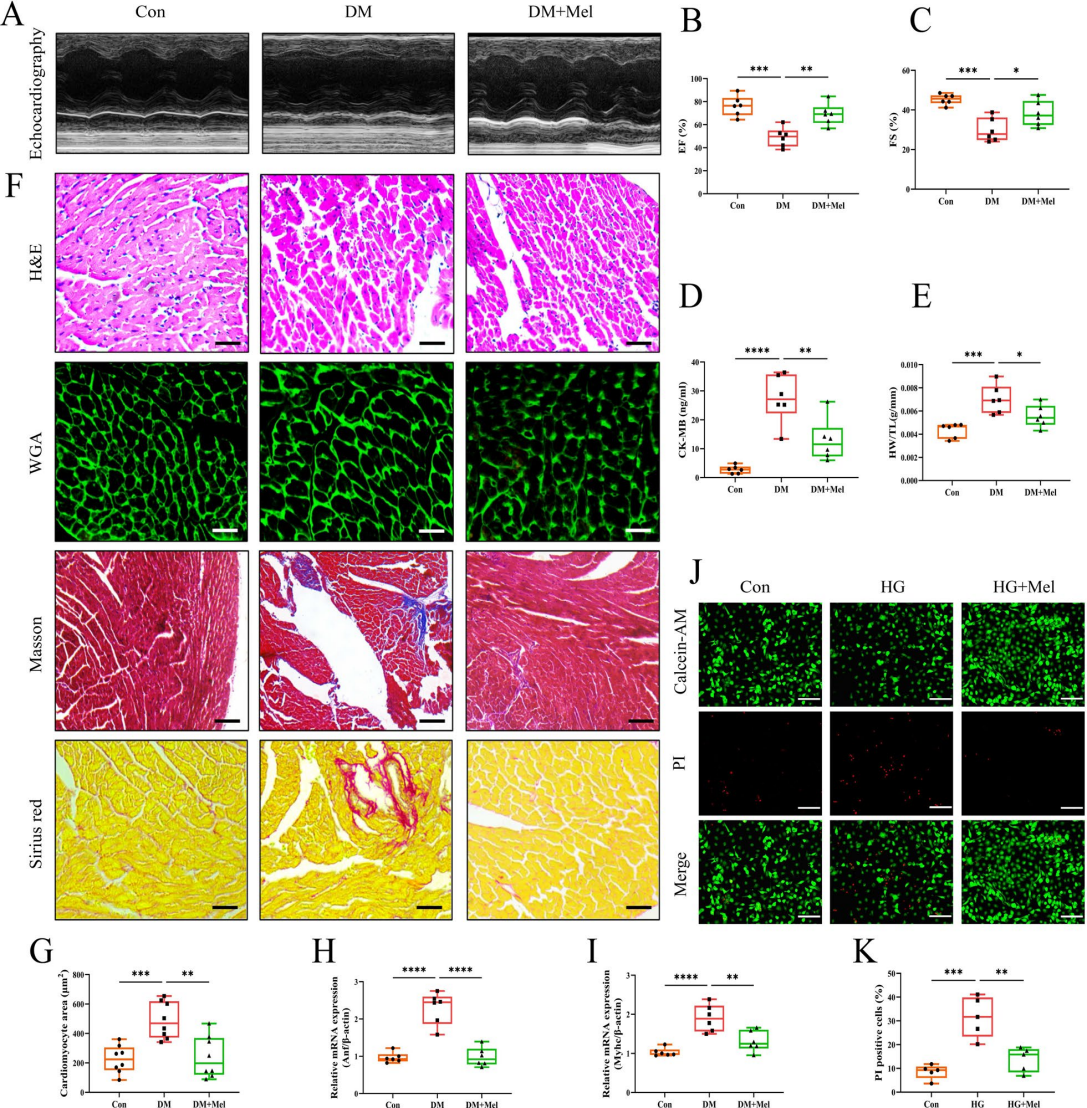
Mel alleviated cardiac dysfunction in diabetic mice. **A** Representative images of echocardiography in mice. **B**, **C** Values of EF and FS, n = 6. **D** CK-MB release in serum, n = 6. **E** Ratio of heart weight to tibia length, n = 6. **F** Myocardial tissues were stained with H&E (scale bar = 50 μm), WGA (scale bar = 50 μm), Masson (scale bar = 100 μm) and Sirius red (scale bar = 50 μm). **G** Size of myocardial cells were assessed by staining with WGA, n = 8. **H**, **I** The mRNA expressions of *Anf* and *Myhc*, n = 6. **J** Calcein-AM/PI double staining in NRVMs (scale bar = 200 μm). **K** Quantification of PI-positive cells, n = 5. Data were expressed as the mean ± SD.*p < 0.05, **p < 0.01, ***p < 0.001, ****p < 0.0001. One-way ANOVA followed by a post hoc Tukey’s test

The experimental protocol for this section was shown in Additional file 1: Fig S1A. We found that Mel treatment did not affect the blood glucose level, glucose tolerance, body weight, food intake and water intake in DM mice (Additional file 1: Fig S1B–H). Echocardiography showed that EF and FS were significantly decreased in DM group, whereas, Mel treatment markedly increased the values of EF and FS when compared with DM group (Fig. 1A–C). Mel treatment significantly reduced serum CK-MB release in comparison with DM group (Fig. 1D). DCM caused higher heart weight/tibia length (HW/TL) ratio, larger cardiomyocyte size with increased *Anf* and *Myhc* mRNA expressions and more fibrosis deposition compared to the Con group, whereas, Mel treatment notably alleviated these above changes (Fig. 1E–I). Calcein-AM/PI staining was done to examine cardiomyocytes live/death in NRVMs. Abundant NRVMs were dead after high glucose (HG) treatment. However, Mel treatment attenuated cell death in comparison with HG group (Fig. 1J, K).

### Mel attenuated DCM through increasing cardiomyocytes autophagy

**Fig. 2:**
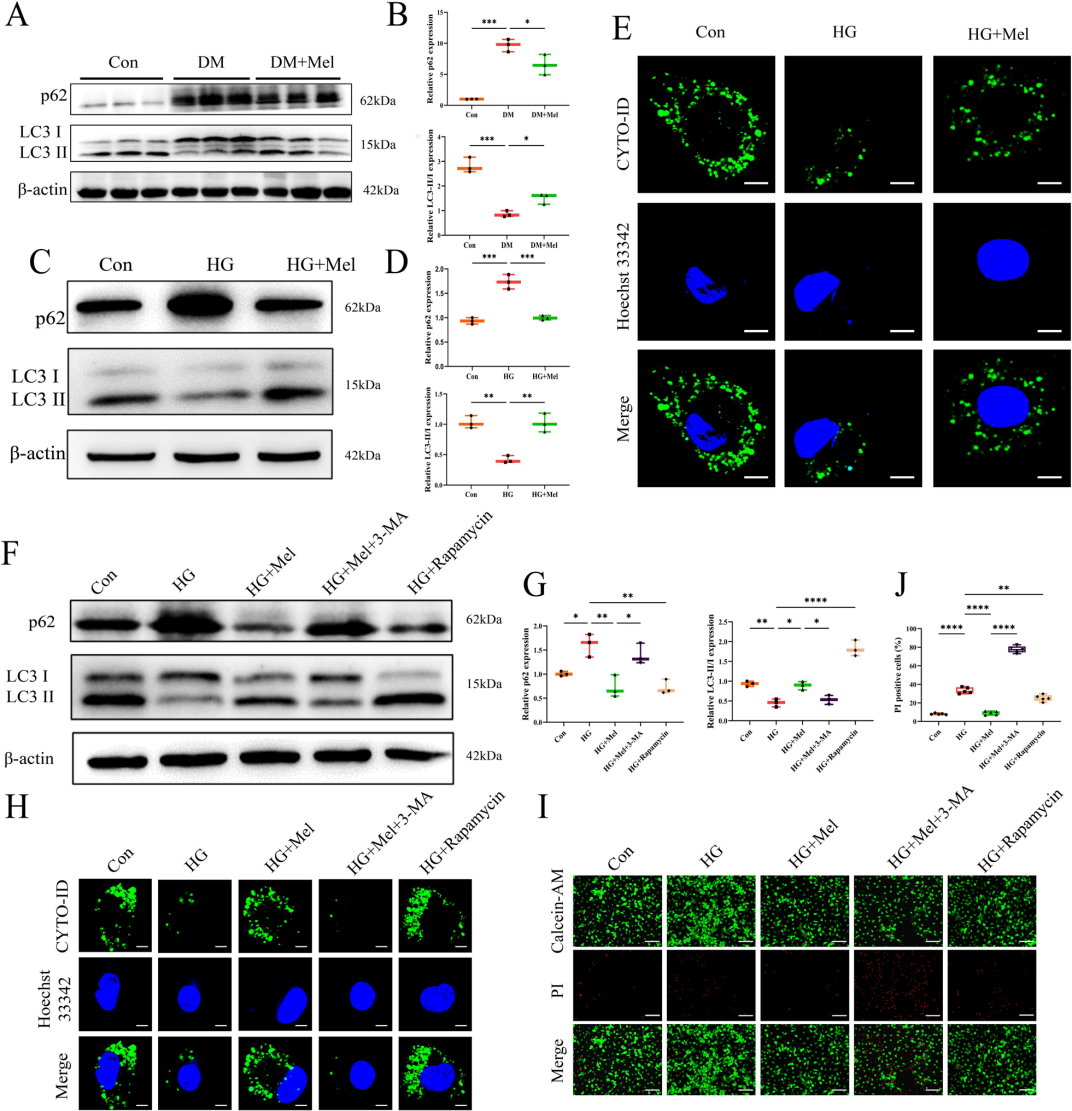
Mel attenuated DCM through increasing cardiomyocytes autophagy. **A** Western blot for p62 and LC3 in mice. **B** Quantification for p62 and LC3-II/LC3-I in mice, n = 3. **C** Western blot for p62 and LC3 in NRVMs. **D** Quantification for p62 and LC3-II/LC3-I in NRVMs, n = 3. **E** Autophagic flux was used to detect autophagy in NRVMs (scale bar = 10 µm). **F** Western blot for p62 and LC3 in NRVMs. **G** Quantification for p62 and LC3-II/LC3-I in NRVMs, n = 3. **H** Autophagic flux in NRVMs (scale bar = 10 µM) **I** Calcein-AM/PI double staining in NRVMs (scale bar = 200 μm) **J** Quantification of PI-positive cells, n = 5. Data were expressed as the mean ± SD.*p < 0.05, **p < 0.01, ***p < 0.001, ****p < 0.0001. One-way ANOVA followed by a post hoc Tukey’s test

Western blot showed down-regulated p62 expression and increased ratio of LC3-II/LC3-I in Mel treatment group mice compared with DM group (Fig. 2A, B). The similar results were also obtained in NRVMs (Fig. 2C, D), indicating that Mel significantly increased cardiomyocytes autophagy either in vivo or in vitro. Additionally, autophagic flux test showed that Mel treatment did enhance autophagy of NRVMs (Fig. 2E). Moreover, autophagy agonist rapamycin treatment showed the similar effect to Mel, while autophagy inhibitor 3-MA treatment neutralized the effect of Mel on NRVMs (Fig. 2F–H). Importantly, calcein-AM/PI staining showed that 3-MA treatment reversed the protective effect of Mel on HG-treated NRVMs (Fig. 2I, J), indicating that Mel attenuated DCM-induced cell death by increasing autophagy of cardiomyocytes.

### Mel down-regulated expression of VEGF-B either in vivo or in vitro

**Fig. 3:**
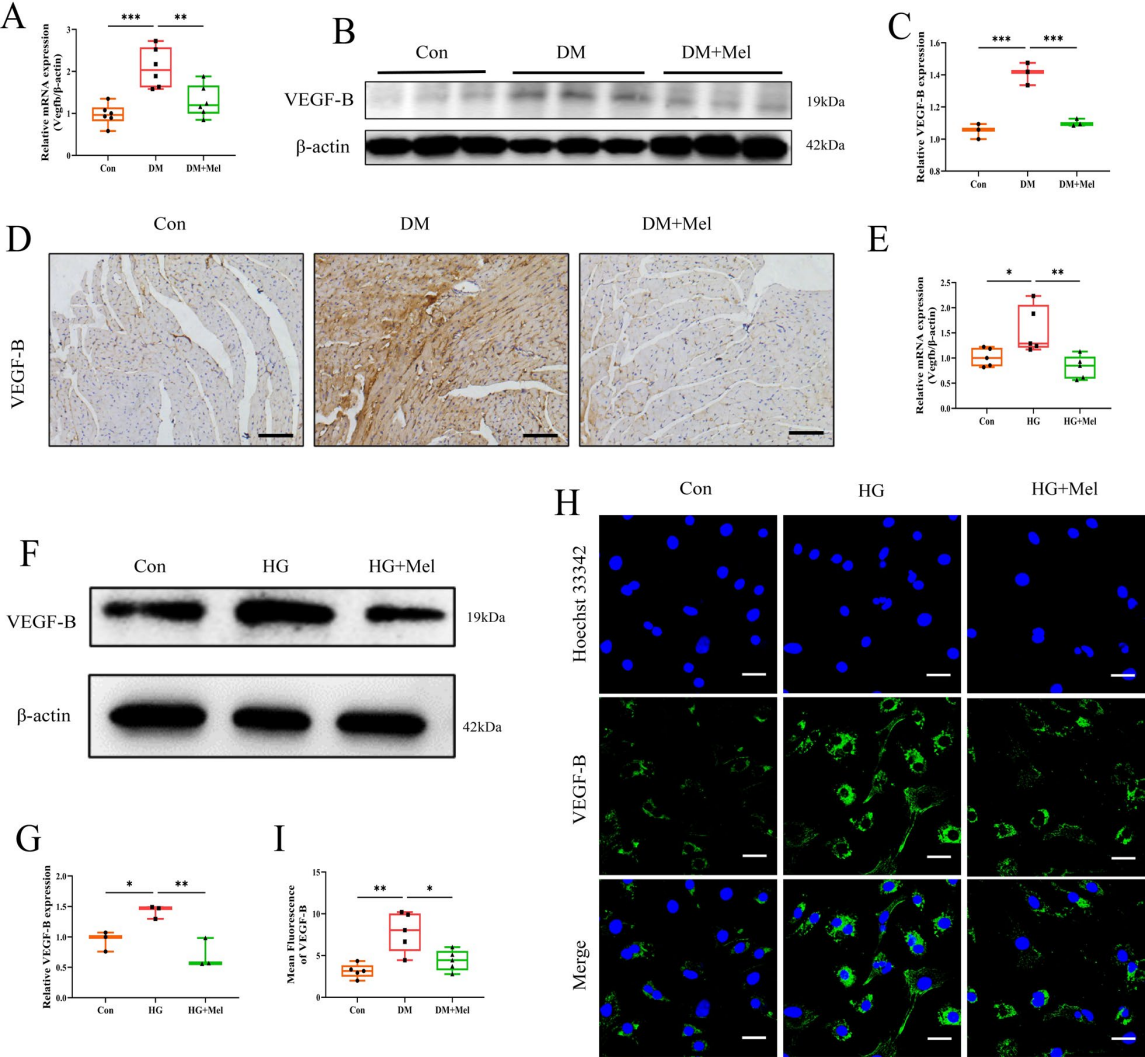
Mel down-regulated expression of VEGF-B either in vivo or in vitro. **A** The mRNA expression of *Vegfb* in mice, n = 6. **B** Western blot for VEGF-B in mice. **C** Quantification for VEGF-B in mice, n = 3. **D** The immunohistochemical analysis for VEGF-B in mice myocardial tissues (scale bar = 100 μm). **E** The mRNA expression of *Vegfb* in NRVMs, n = 5. **F** Western blot for VEGF-B in NRVMs. **G** Quantification for VEGF-B in NRVMs, n = 3. **H** The NRVMs were subjected to immunofluorescence analysis with anti-VEGF-B antibody (scale bar = 100 μm). **I** Quantification for mean fluorescence of VEGF-B in NRVMs, n = 5. Data were expressed as the mean ± SD.*p < 0.05, **p < 0.01, ***p < 0.001. One-way ANOVA followed by a post hoc Tukey’s test

We found that Mel treatment decreased the mRNA and protein expressions of VEGF-B in mice heart in comparison with DM group (Fig. 3A-C). Increased VEGF-B expression was found in DM group, and decreased VEGF-B positive areas were observed in Mel treated heart tissues (Fig. 3D). Similarly, Mel decreased the mRNA (Fig. 3E) and protein levels (Fig. 3F, G) of VEGF-B in NRVMs. The results of immunofluorescence test for VEGF-B showed similar tendency with the western and real-time PCR results (Fig. 3H, I). These data indicated that Mel could reduce expression of VEGF-B in mice with DCM and HG-treated NRVMs.

### VEGF-B overexpression abolished the cardioprotective effect of Mel on DCM

**Fig. 4:**
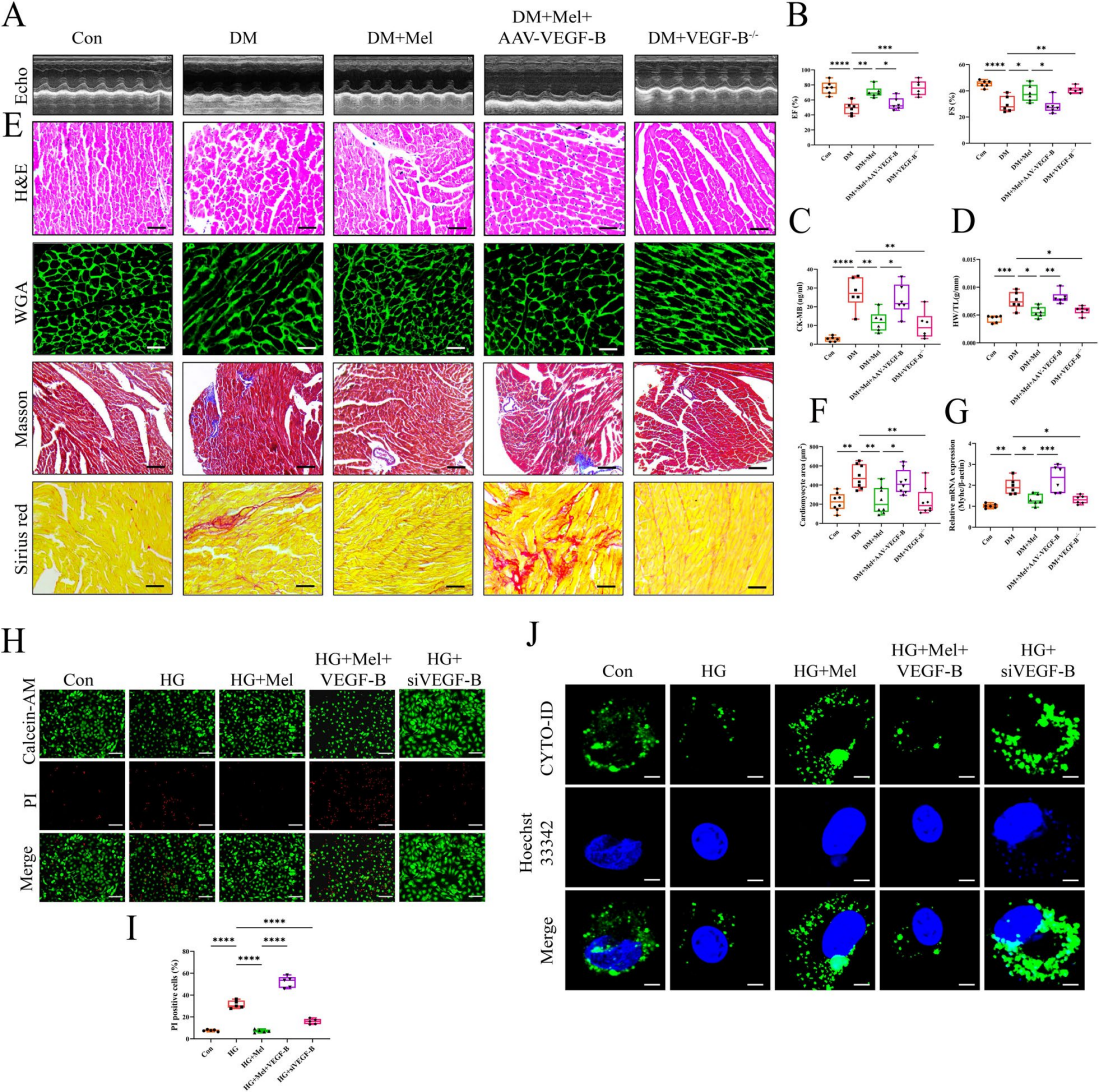
VEGF-B overexpression abolished the cardioprotective effect of Mel on DCM. **A** Representative images of echocardiography in mice. **B** Values of EF and FS, n = 6. **C** CK-MB release in serum, n = 6. **D** Ratio of heart weight to tibia length, n = 6. **E** Myocardial tissues were stained with H&E (scale bar = 50 μm), WGA (scale bar = 50 μm), Masson (scale bar = 100 μm) and Sirius red (scale bar = 50 μm). **F** Size of myocardial cells were assessed by staining with WGA, n = 8. **G** The mRNA expression of *Myhc*, n = 6. **H** Calcein-AM/PI double staining in NRVMs (scale bar = 200 μm). **I** Quantification of PI-positive cells, n = 5. **J** Autophagic flux in NRVMs (scale bar = 10 µm). Data were expressed as the mean ± SD.*p < 0.05, **p < 0.01, ***p < 0.001, ****p < 0.0001. One-way ANOVA followed by a post hoc Tukey’s test

To further investigate the role of VEGF-B in Mel inducing cardio-protection on DCM, we used VEGF-B global knock out (VEGF-B^−/−^) mice and VEGF-B overexpressed (AAV-VEGF-B) mice in the following study. After the genotype identification of VEGF-B knockout mice by PCR, the mRNA levels of VEGF-B in WT, VEGF-B^+/−^ and VEGF-B^−/−^ mice were detected by qPCR (Additional file 1: Fig S2A, B). To verify the efficiency of AAV-VEGF-B, we detected mRNA and protein levels of VEGF-B at 4 weeks (Additional file 1: Fig S2C, D). Meanwhile, the expression of VEGF-B in heart tissue were also detected by immunohistochemical staining (Additional file 1: Fig S2E). The in vivo experimental protocol for this section was shown in Additional file 1: Fig S2F. In comparison with DM group, DM + VEGF-B^−/−^ group exhibited extremely lower VEGF-B mRNA level. Conversely, in comparison with DM + Mel group, DM + Mel + AAV-VEGF-B group showed significantly higher VEGF-B mRNA level (Additional file 1: Fig S2G). The metabolic parameters for each group were shown in Additional file 1: FigS3A–F, we found that VEGF-B knock out and AAV-VEGF-B did not change the blood glucose level, glucose tolerance, body weight, food intake and water intake in DM mice. As shown in Fig. 4A, B, VEGF-B^−/−^ DM mice and Mel treatment caused higher EF and FS when compared with WT DM mice. However, AAV-VEGF-B injection resulted in decreased EF and FS in comparison with Mel single treatment. Serum CK-MB levels were significantly increased by AAV-VEGF-B administration despite Mel treatment but reduced in VEGF-B^−/−^ DM mice (Fig. 4C). Mel treatment significantly reduced HW/TL ratio, cardiomyocytes areas and expression of *Myhc*, namely inhibited DCM-induced myocardial hypertrophy. However, overexpression of VEGF-B markedly abolished these above positive effects of Mel. Interestingly inhibition of VEGF-B exhibited the similar effect of Mel on myocardial hypertrophy and fibrosis (Fig. 4D–G). Simultaneously, VEGF-B siRNA obviously reduced cardiomyocytes death in NRVMs, whereas, VEGF-B overexpression neutralized the protective effect of Mel (Fig. 4H, I). Noteworthy, Mel failed to increase cardiomyocytes autophagy in the AAV-VEGF-B group, but inhibition of VEGF-B well enhanced autophagy either in DM mice or NRVMs (Fig. 4J, Additional file 1: Fig S4A, B). Interestingly, our findings suggested that under physiological conditions, overexpression or deletion of VEGF-B did not impact autophagy (Additional file 1: Fig S4C, D).

### VEGF-B interacted with GRP78 in NRVMs

**Fig. 5:**
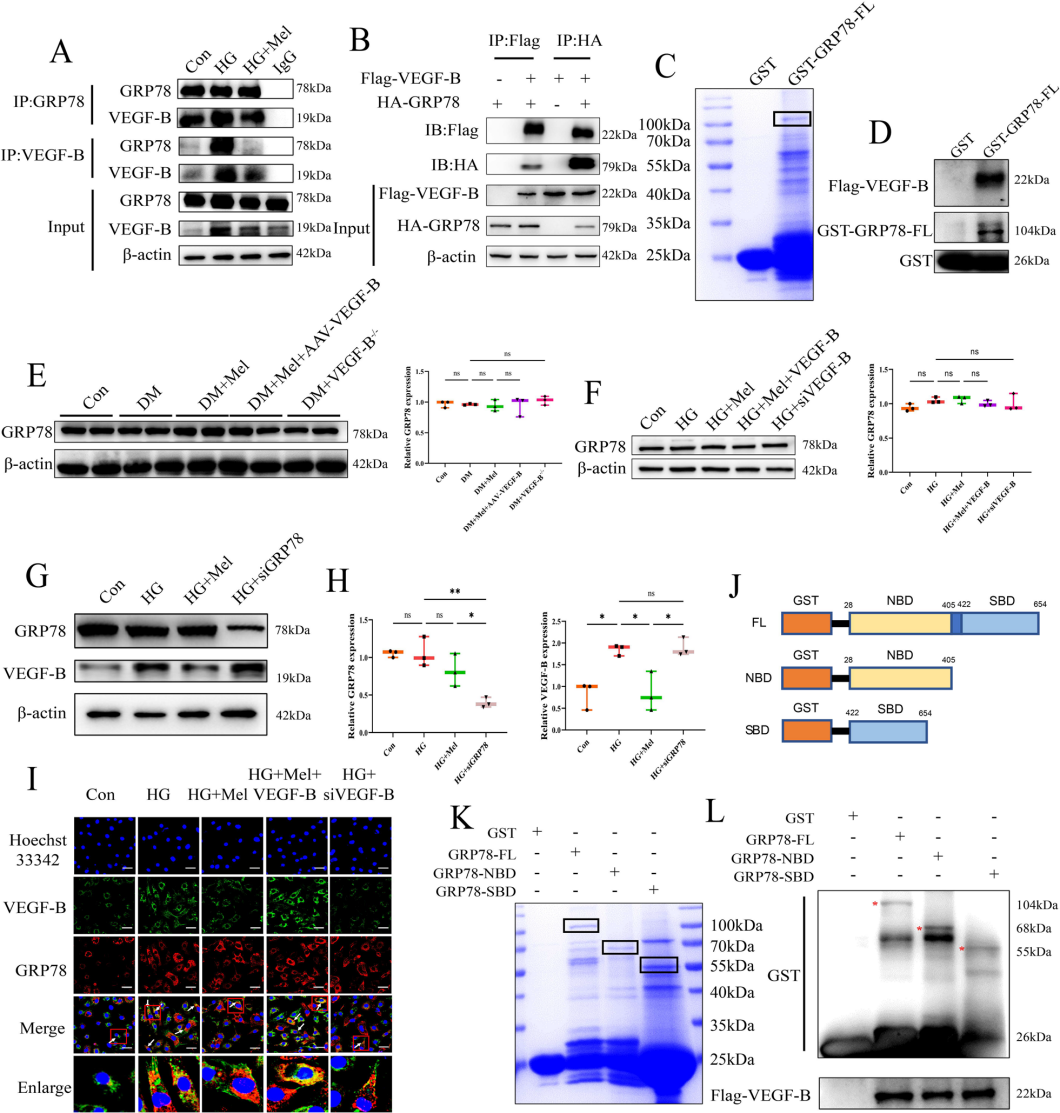
VEGF-B interacted with GRP78 in NRVMs. **A** Co-immunoprecipitation was performed to detect the interaction of VEGF-B and GRP78 in NRVMs and (**B**) in 293 T cells. **C** We constructed GST-GRP78-FL plasmid, expressed and purified in Rosetta (DE3). **D** Co-incubated with Flag-VEGF-B to detect the interaction between GRP78 and VEGF-B. **E** Western blot for GRP78 and quantification for GRP78 in mice, n = 3. **F** Western blot for GRP78 and quantification for GRP78 in NRVMs, n = 3. **G** Western blot for GRP78 and VEGF-B in NRVMs. **H** Quantification for GRP78 and VEGF-B in NRVMs, n = 3. **I** Immunofluorescence staining for VEGF-B, GRP78, and their co-localization in NRVMs (scale bar = 50 μm). **J** Different truncates of GRP78. **K** We established and purified GST-GRP78-FL, GST-GRP78-NBD and GST-GRP78-SBD in Rosetta (DE3). **L** VEGF-B interacted with SBD domain, NBD domain and FL of GRP78. Data were expressed as the mean ± SD.*p < 0.05, **p < 0.01. One-way ANOVA followed by a post hoc Tukey’s test

We performed immunoprecipitation-mass spectrometer (IP-MS) in NRVMs to seek proteins which interacted with VEGF-B (Additional file 1: Fig S5A). Through analyzing the IP-MS data, GRP78 was selected to be studied (Additional file 1: Fig S5B). We found that VEGF-B interacted with GRP78 either in NRVMs (Fig. 5A) or in 293 T cells (Fig. 5B). Interestingly, immunoprecipitation assays showed that the interaction between VEGF-B and GRP78 in Mel-treated NRVMs was weaker than that in HG treated cells. In GST pull down assay, GST, GST-GRP78-FL were cloned and purified (Fig. 5C). In vitro experiments demonstrated that Flag-VEGF-B protein was pulled down by GST-GRP78-FL (Fig. 5D), indicating that there existed an interaction between VEGF-B and GRP78.

Then, we found that inhibition or overexpression of VEGF-B did not change the expression of GRP78 either in mice (Fig. 5E) or in NRVMs (Fig. 5F). Likewise, inhibition of GRP78 did not change the expression of VEGF-B in NRVMs (Fig. 5G, H). Immunofluorescence staining showed that in comparison with HG group, Mel or VEGF-B siRNA treatment significantly reduced the co-localization of GRP78 and VEGF-B. However, VEGF-B overexpression resulted in increased co-localization of GRP78 and VEGF-B when compared with HG + Mel group (Fig. 5I). Above results indicated that Mel down-regulated the expression of VEGF-B, then reduced the interaction of VEGF-B and GRP78, thereby increased autophagy of cardiomyocytes. And this process was not dependent on the changes of GRP78 expression.

GRP78 assisted in a wide range of protein folding processes via its two structural domains. One is nucleotide-binding domain (NBD) and the other is substrate-binding domain (SBD) [33]. Under physiological conditions, the NBD of GRP78 binds to PERK and the SBD binds to misfolded proteins. GRP78 binds to the PERK via its NBD and its release is dependent upon misfolded proteins binding to SBD of GRP78 [34]. To further explore the interaction between VEGF-B and GRP78, we constructed truncates from two important domains of GRP78 (Fig. 5J). GST-GRP78-FL, GST-GRP78-NBD and GST-GRP78-SBD were cloned and purified (Fig. 5K). We found that VEGF-B interacted with GRP78 not only in SBD, but also in NBD of GRP78 (Fig. 5L), indicating that the interaction of these two proteins is not due to the VEGF-B misfolding.

### Mel enhanced autophagy and protected against DCM via increasing phosphorylation of PERK

**Fig. 6:**
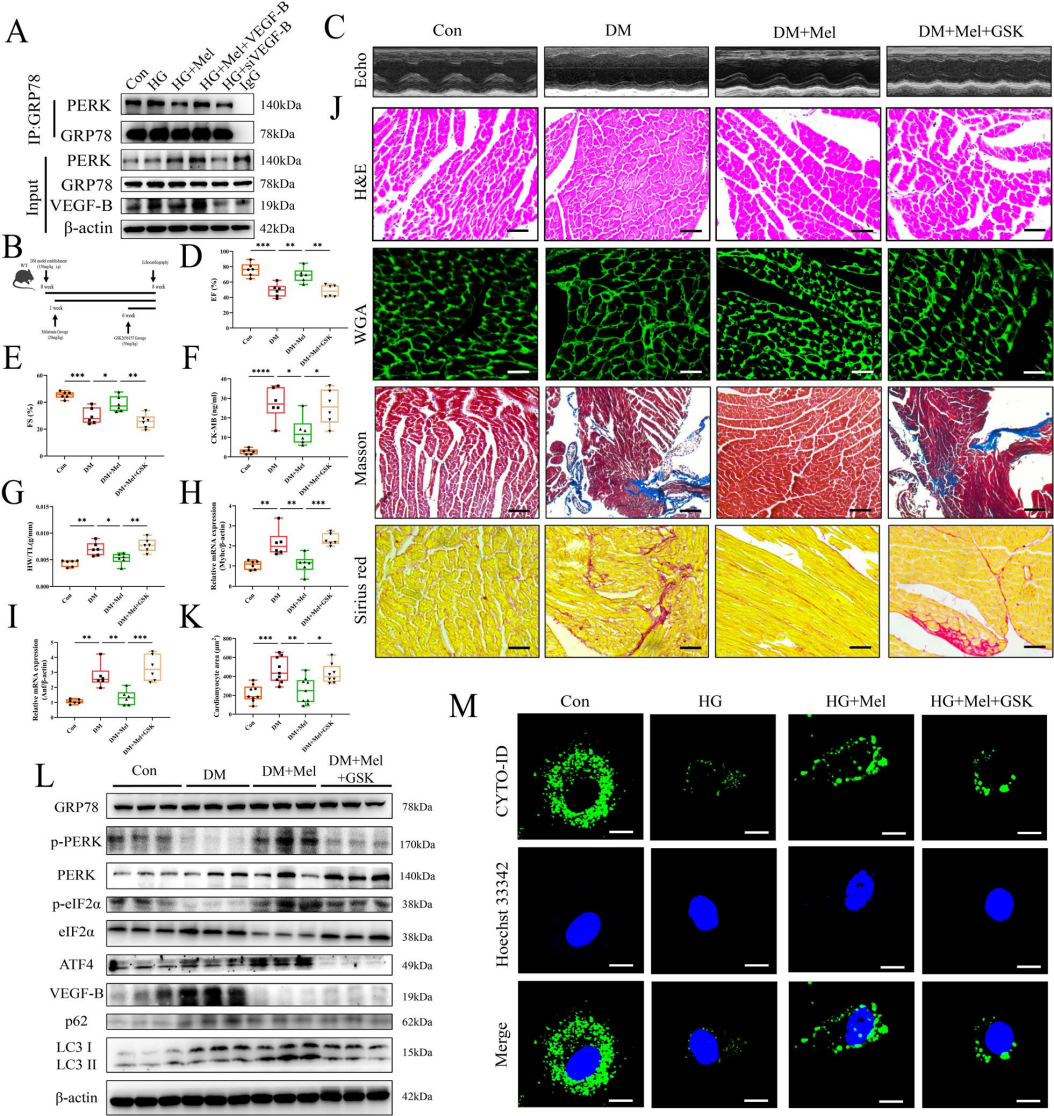
Mel enhanced autophagy and protected against DCM via increasing phosphorylation of PERK. **A** Co-immunoprecipitation was performed to detect the interaction of PERK and GRP78 in NRVMs. **B** The experimental protocol in mice. **C** Representative images of echocardiography in mice. **D** Values of EF and (**E**) FS, n = 6. **F** CK-MB release in serum, n = 6. **G** Ratio of heart weight to tibia length, n = 6. **H**, **I** The mRNA expressions of *Myhc* and *Anf*, n = 6. **J** Myocardial tissues were stained with H&E (scale bar = 50 μm), WGA (scale bar = 50 μm), Masson (scale bar = 100 μm) and Sirius red (scale bar = 50 μm). **K** Size of myocardial cells were assessed by staining with WGA, n = 8. **L** Western blot for PERK-related pathways and autophagy proteins mice, n = 3. **M** Autophagic flux in NRVMs (scale bar = 10 µm) Data were expressed as the mean ± SD. *p < 0.05, **p < 0.01, ***p < 0.01, ****p < 0.0001. One-way ANOVA followed by a post hoc Tukey’s test

It has been demonstrated that GRP78 binds to PERK, and the separation from GRP78 results in autophosphorylation of PERK, thereby initiates eIF2α/ATF4 regulated autophagy [35]. In comparison with HG treatment group, Mel treatment or inhibition of VEGF-B by siRNA reduced the interaction of GRP78 and PERK in NRVMs. However, VEGF-B overexpression caused tighter interaction of GRP78 and PERK than Mel treatment group (Fig. 6A). We subsequently administrated GSK, an inhibitor of phosphorylated PERK, to the mice (Fig. 6B). GSK treatment did not change the metabolic parameters of mice (Additional file 1: Fig S6A-G). We found that GSK treatment significantly attenuated the protective effect of Mel on cardiac function in DCM mice (Fig. 6C–F). And Mel failed to attenuate cardiac hypertrophy and fibrosis in GSK treated mice (Fig. 6G–K). Mel augmented the phosphorylation of PERK and up-regulated the expression of phosphorylated eIF2α and activating ATF4, thereby inducing autophagy in DCM. Conversely, the ability of Mel to induce downstream activation of the PERK pathway and increase autophagy was nullified when PERK phosphorylation was suppressed in diabetic mice and NRVMs. (Fig. 6L, M, Additional file 1: Fig S6H). Interestingly, administration of GSK did not affect the expression of VEGF-B and GRP78. GSK treatment also significantly increased cardiomyocytes death when compared to the Mel alone treatment in NRVMs (Additional file 1: Fig S6I, J).

### Mel attenuated DCM through VEGF-B/ PERK signaling pathway

**Fig. 7:**
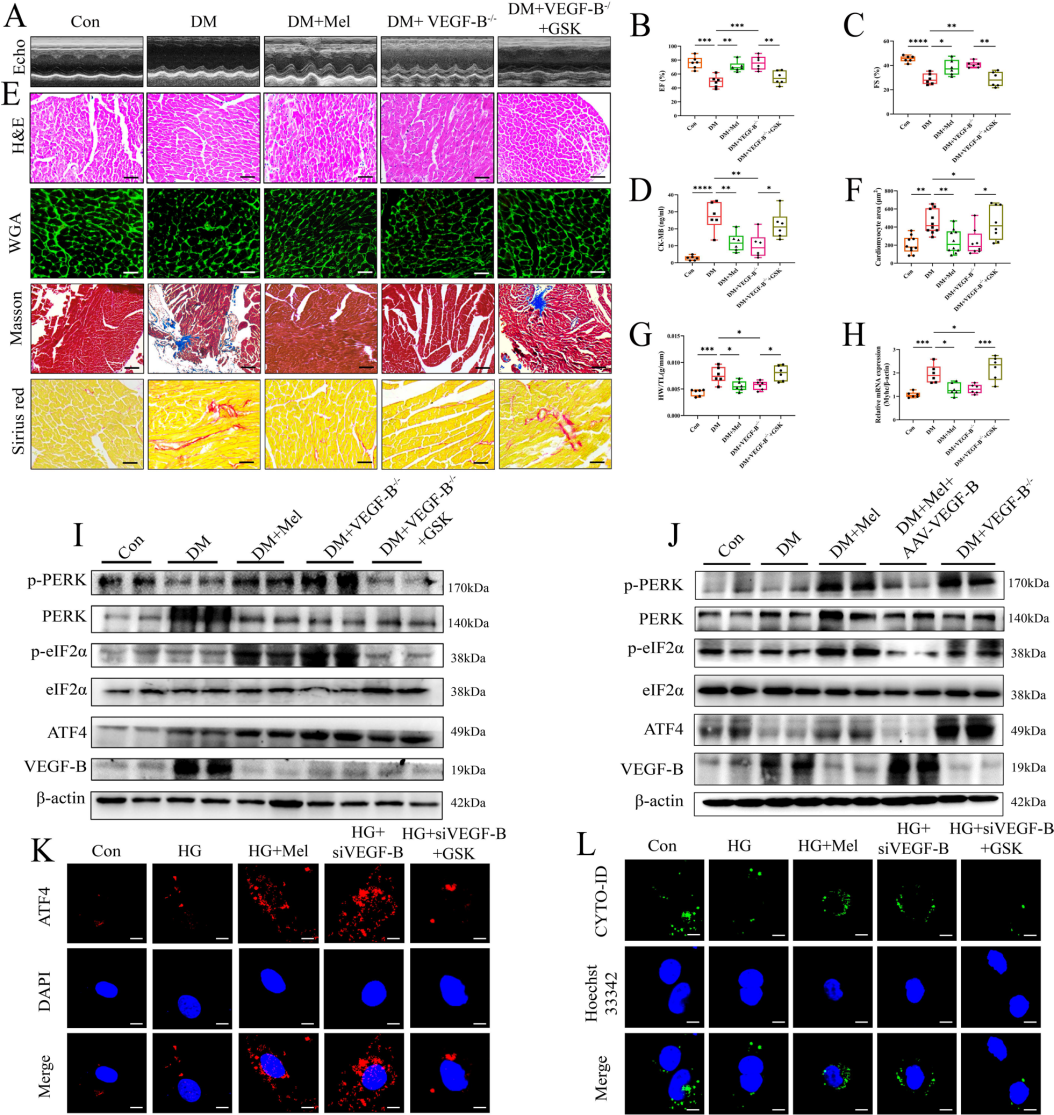
Mel attenuated DCM through VEGF-B/PERK signaling pathway. **A** Representative images of echocardiography in mice. **B** Values of EF and (**C**) FS, n = 6. **D** CK-MB release in serum, n = 6. **E** Myocardial tissues were stained with H&E (scale bar = 50 μm), WGA (scale bar = 50 μm), Masson (scale bar = 100 μm) and Sirius red (scale bar = 50 μm). **F** Size of myocardial cells were assessed by staining with WGA, n = 8. **G** Ratio of heart weight to tibia length, n = 6. **H** The mRNA expression of *Myhc*, n = 6. **I** Western blot for PERK-related pathways and autophagy proteins in GSK treated mice, n = 3. **J** Western blot for PERK-related pathways and autophagy proteins in mice, n = 3. **K** Immunofluorescence staining for ATF4 translocation in NRVMs (scale bar = 10 μm). **L** Autophagic flux in NRVMs (scale bar = 10 μm). Data were expressed as the mean ± SD.*p < 0.05, **p < 0.01, ***p < 0.01, ****p < 0.0001. One-way ANOVA followed by a post hoc Tukey’s test

To investigate the relationship between VEGF-B and PERK in DCM, the following experiments were conducted (Additional file 1: Fig S7A). The metabolic parameters of mice were shown in Additional file 1: Fig S7B–H, and echocardiography was performed (Fig. 7A). VEGF-B^−/−^ DM mice showed higher LV function than WT DM mice, but administration of GSK reduced EF and FS in VEGF-B^−/−^ DM mice (Fig. 7B, C). Serum CK-MB levels were significantly reduced in VEGF-B^−/−^ diabetic mice but increased after GSK treatment (Fig. 7D). Additionally, GSK treatment reversed the VEGF-B deletion effect on attenuating myocardial hypertrophy and fibrosis in DM mice (Fig. 7E–H). We found that GSK treatment significantly reduced phosphorylation of PERK and eIF2α with down-regulated expression of ATF4 in mice even though VEGF-B was knocked out (Fig. 7I). Meanwhile, we found that VEGF-B knockout significantly activated the PERK signaling pathway, namely, increased phosphorylation of PERK and eIF2α. However, overexpression of VEGF-B inhibited the PERK signaling pathway in mice (Fig. 7J). It has been reported that ATF4 can translocate into the nucleus to increase the transcriptional level of LC3 [36]. The mRNA levels of *Atf4* were increased either in Mel treatment group or in VEGF-B knock out group. Conversely, increased mRNA levels of *Atf4* were observed in the GSK treatment and AAV-VEGF-B group. (Additional file 1: Fig S7I, J). Then, we examined the translocation of ATF4 by immunofluorescence staining in NRVMs. We found that the translocation of ATF4 increased after Mel treatment or VEGF-B deletion, but GSK treatment prevented nucleus translocation of ATF4 (Fig. 7K). Furthermore, autophagy was reduced with GSK treatment in VEGF-B knockdown group when compared to VEGF-B knockdown group (Fig. 7L). All the results suggested that Mel attenuated DCM through VEGF-B/PERK signaling pathway mediated autophagy.

## Discussion

T1DM and type 2 diabetes mellitus (T2DM) had distinct impacts on the left ventricular function and myocardial performance. In T1DM, pronounced apoptotic cardiomyocyte deaths, reactive hypertrophy, fibrosis, elevated cardiac oxidative stress, DNA damage, and senescence were observed compared to T2DM in mice. In conclusion, the T1DM and T2DM mice models showed significant differences in cardiac remodeling, function, and the overall transcriptome [37]. Mel exhibited cardio-protective effect in previous studies [7]. However, the molecular mechanism of T1DM induced DCM was fully unknown. Basing on the protocol of previous study, the dose of Mel in animal experiments was set to 20 mg/kg [9]. And we found that Mel treatment significantly improved cardiac function in DCM (Fig. 1) without changes on metabolic parameters in diabetic mice (Additional file 1: Fig S1B–H), indicating that other molecular mechanisms might be involved in cardio-protective effect of Mel on DCM.

Autophagy plays dual roles in cardiovascular diseases. Loss of autophagy exacerbated Ang-II-induced cardiac hypertrophy, linked to increased ROS production and NF-κB activation in macrophages [38]. In contrast, during myocardial ischemia/reperfusion injury, the size of myocardial infarction area was worsened after autophagy restoration [39]. Importantly, the roles of autophagy in DCM are controversial. One study showed that neuregulin-4 attenuates diabetic cardiomyopathy by regulating autophagy via the AMPK/mTOR [40]. Conversely, overexpression of Beclin1 aggravated diabetes-induced cardiac abnormalities in type 1 diabetes [41]. Mel has been reported to regulate autophagy. Fatemeh Y et al. reported that Mel ameliorates arsenic-induced cardiotoxicity by decreasing autophagy [42]. However, Mel protected against sepsis-induced cardiac dysfunction by increasing autophagy via activation of SIRT1 [43]. Therefore, it is essential to confirm the role of autophagy on Mel treatment in DCM. In present study, we found that Mel treatment significantly increased autophagy of cardiomyocytes either in mice or in NRVMs and an inhibitor of autophagy 3-MA, reversed the protective effect of Mel on cardiomyocytes in HG-treated NRVMs (Fig. 2), indicating that Mel attenuated DCM by increasing autophagy of cardiomyocytes.

Likewise, VEGF-B plays different roles in a variety of diseases. VEGF-B exhibited different effects on angiogenesis dependent on the extent of its binding to FGF/FGFR1 in cancer [44]. Our previous study showed that up-regulation of VEGF-B protects against myocardial ischemia–reperfusion injury by decreasing oxidative stress [45]. However, suppression of cardiac VEGF-B following myocardial infarction implies its potential insignificance in cardiac repair [46]. Recent study discovered that the overexpression of VEGF-B potentially induced hypertrophy of the myocardium [47]. And, Hagberg et al. reported that inhibition of VEGF-B increased muscle lipid uptake and it was identified as a target for the treatment of type 2 diabetes [15]. Interestingly, VEGF-B was significantly highly expressed in diabetic nephropathy [48] and accelerated the development of pathological neovascularization in diabetic retinopathy [49]. The relationship between Mel and VEGF-B has not been reported yet, and the role of VEGF-B in autophagy remains unknown. In present study, we found that VEGF-B was highly expressed in DCM mice and HG treated NRVMs, while Mel decreased the VEGF-B mRNA and protein levels (Fig. 3). Furthermore, overexpression of VEGF-B significantly abolished the protective effect of Mel on DCM and autophagy either in vivo or in vitro (Fig. 4), indicating that Mel protected against DCM via VEGF-B regulated autophagy.

To find out how VEGF-B regulated autophagy in DCM, IP-MS was performed. GRP78 was found to have interaction with VEGF-B (Additional file 1: Fig S5A, B; Fig. 5A–H). GRP78 is an important chaperone in ER and involved in the activation of ER stress through the UPR pathway. The UPR functions in coping with protein-folding stress through three pro-survival mechanisms, which was activated by at least three stress sensors, IRE1α, PERK and ATF6. And PERK phosphorylates eIF2α to decrease overall translation while increasing specific translation of genes, including ATF4 [50].

However, we found that Mel treatment and inhibition of VEGF-B by siRNA did not change the expression of GRP78, indicating the cardio-protective of Mel is not dependent on the expression of GRP78. GRP78 has two structural domains, namely NBD and SBD. Under physiological conditions, proteins in ER bind to SBD, which greatly stimulates ATPase activity within NBD, enabling GRP78 to adopt an ADP-bound (low K-on and K-off) closed conformation which traps misfolded protein substrate, then inducing the UPR pathway [35]. Does VEGF-B just serve as misfolded protein and only bind to SBD of GRP78? Interestingly, we found that VEGF-B not only bound to SBD but also NBD of GRP78 (Fig. 5J–L), indicating that VEGF-B did not serve as a misfolded protein to bind to GRP78, and this binding of VEGF-B and GRP78 probably changed the protein conformation of GRP78, then resulted in the changes of their down-stream genes. More importantly, in comparison with HG group, Mel treatment significantly reduced the interaction and co-localization between VEGF-B and GRP78 (Fig. 5A, I). Therefore, we speculated that Mel treatment down-regulated the expression of VEGF-B, then reduced the interaction of VEGF-B and GRP78, thereafter initiated the following signal pathway.

**Fig. 8:**
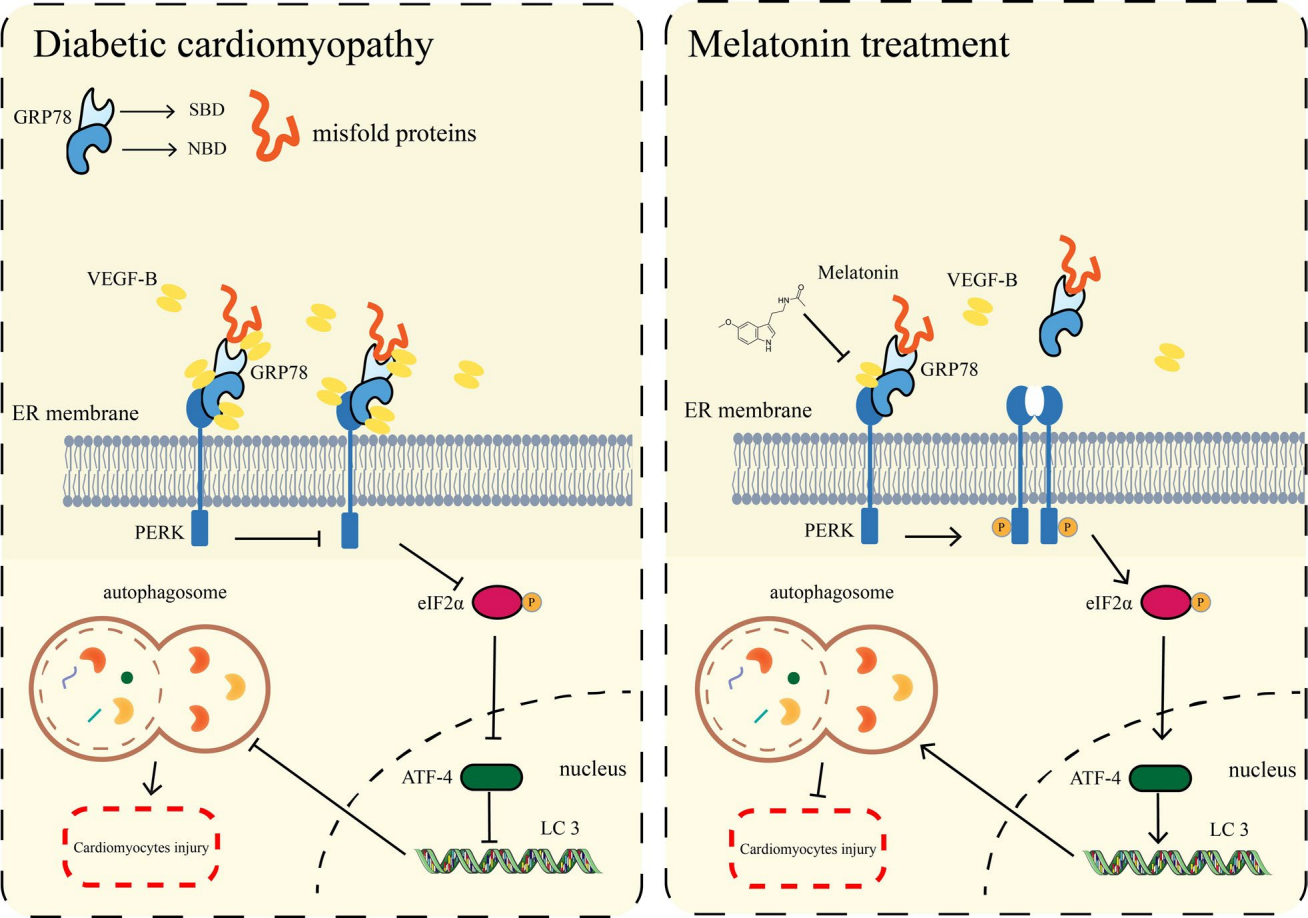
The overview diagram of this study. Mel attenuated DCM by increasing autophagy of cardiomyocytes via regulation of VEGF-B/GRP78/PERK signaling pathway

ER stress and UPR activation have important roles in the pathogenesis of many diseases. UPR is used to maintain an optimal rate of protein production and respond quickly to various stimuli [51]. ER stress functions as a double-edged sword, with deficiency of ER stress resulting in cellular defects causing disturbed cardiovascular function. However, if ER stress is severe, the UPR may stimulate apoptosis [16]. Therefore, a moderate but not excessive level of ER stress is necessary for cell function. In general, PERK/eIF2α/ATF4 pathway is essential for autophagy induction after ER stress. Therefore, we investigated the mechanism of VEGF-B on the PERK signaling pathway. In previous study, deletion of PERK in Thbs1 transgenic mice largely corrected the lethal cardiac atrophy [25].and inhibition of PERK had a neuroprotective effect, but impaired glycemic control [52]. In contrast, Piccolis M et al. demonstrated that inhibition of the PERK appeared to aggravate the detrimental effects of palmitate [53]. Long-term activation of PERK could help cells counteract protein misfolding and promoted cell survival or death under chronic or severe ER stress conditions [54]. In recent study, ablation of PERK activity in human and rodent β-cells impairs the synthesis of insulin [55, 56]. We found that GSK, an inhibitor of phosphorylation of PERK significantly abolished the protective effect of Mel with decreased autophagy level (Fig. 6). Furthermore, overexpression of VEGF-B markedly reduced the phosphorylation of PERK and eIF2α (Fig. 7). Taken together, we drew the conclusion that Mel attenuated DCM by increasing the autophagy of cardiomyocytes, and this cardio-protective effect depended on VEGF-B/GRP78/PERK signaling pathway (Fig. 8).

## Conclusions

To our knowledge, this is the first time that the relationship between Mel treatment and VEGF-B expression during DCM is revealed. Therefore, Mel can be used as a potential cardio-protective adjuvant in DCM therapy and down-regulation of VEGF-B/GRP78/PERK signaling pathway will be a promising modality for clinical DCM therapy.

### Supplementary Information

**Additional file 1: Figure S1.** The metabolic parameters for WT mice. (A) The experimental protocol in WT mice. (B-C) Fasting and non-fasting blood glucose levels, n=8. (D) IPGTT was performed at week 8, n=6. (E) Area of under curve (AUC) of IPGTT was calculated, n=6. (F-H) Body weight, food intake and water intake were obtained, n=8. Data were expressed as the mean ± SD. ***p<0.001, ****p<0.0001. One-way ANOVA followed by a post hoc Tukey’s test. **Figure S2.** Construction of global VEGF-B knock out mice and AAV-VEGF-B overexpression mice. (A) PCR analysis of VEGF-B genotype for the presence of WT, heterozygotes (vegf-b+/-) and homozygous (vegf-b-/-). (B) mRNA levels of Vegfb in WT, VEGFB^+/-^ and VEGF-B^-/-^ mice, n=5. (C) mRNA levels of *Vegfb* in WT, Vector and AAV-VEGF-B mice, n=5. (D) We detected Flag-tag in AAV injection mice heart by western blot. (E) The immunohistochemical analysis for VEGF-B in mice myocardial tissues (scale bar=100μm). (F) The experimental protocol for WT VEGF-^B-/-^ and AAV-VEGF-B mice. (G) mRNA levels of * Vegfb* in mice, n=5. Data were expressed as the mean ± SD. ****p<0.0001. One-way ANOVA followed by a post hoc Tukey’s test. **Figure S3.** The metabolic parameters for WT, VEGF-B^-/-^ and AAV-VEGF-B mice. (A, B) fasting and none-fasting blood glucose, n=8. (C) IPGTT was performed at week 8, n=6. (D) AUC of IPGTT was calculated, n=6. (E-F) Body weight, food intake and water intake were obtained, n=8. Data were expressed as the mean ± SD. ***p<0.001, ****p<0.0001. One-way ANOVA followed by a post hoc Tukey’s test. **Figure S4.** The absence of VEGF-B or AAV-VEGF-B did not affect the autophagy in normal mice. (A) Western blot for VEGF-B, p62 and LC3 in mice. Quantification for p62, VEGF-B and LC3 II/I in mice, n=3. (B) Western blot for VEGF-B, p62 and LC3 in NRVMs. Quantification for p62, VEGF-B and LC3 II/I in NRVMs, n=3. (C) Western blot for VEGF-B, p62 and LC3 II/I in VEGF-B-/- and AAV-VEGF-B mice in mice heart by western blot, n=3. (D) Quantification for p62, VEGF-B and LC3 II/I, n=3. Data were expressed as the mean ± SD. *p<0.05, **p<0.01, ***p<0.01, ****p<0.0001. One-way ANOVA followed by a post hoc Tukey’s test. **Figure S5.** IP-MS in NRVMs with high glucose treatment to find VEGF-B interactors. (A) Coomassie brilliant blue staining. (B) The candidates for VEGF-B interactors. **Figure S6.** The metabolic parameters for GSK treated mice. (A, B) Fasting and none-fasting blood glucose, n=8. (C) IPGTT was performed at week 8, n=6. (D) AUC of IPGTT was calculated, n=6. (E-G) Body weight, food intake and water intake were obtained, n=8. (H) Quantification for PERK-related pathways and autophagy proteins in mice, n=3. (I) Calcein-AM/PI double staining in NRVMs (scale bar=200μm). (J) Quantification of PI-positive cells, n=5. Data were expressed as the mean ± SD. *p<0.05, **p<0.01, ***p<0.001, ****p<0.0001. One-way ANOVA followed by a post hoc Tukey’s test. **Figure S7.** The metabolic parameters in mice. (A) The experimental protocol for VEGF-B^-/-^ and GSK treatment mice. (B, C) Fasting and none-fasting blood glucose, n=8. (D) IPGTT was performed at week 8, n=6. (E) AUC of IPGTT was calculated, n=6. (F-H) Body weight, food intake and water intake were obtained, n=8. (I, J) mRNA levels of *Atf4* in AAV-VEGF-B, VEGF-B^-/-^ and GSK treatment mice, n=6. Data were expressed as the mean ± SD. *p<0.05, **p<0.01, ***p<0.001, ****p<0.0001. One-way ANOVA followed by a post hoc Tukey’s test.

**Additional file 2: Table S1.** Genotyping of WT, VEGF-B^+/-^ and VEGF-B^-/-^.

**Additional file 3: Table S2.** Echocardiographic parameters in mice.

**Additional file 4: Table S3.** List of primer sequences for Realtime-PCR used in this study.

## Data Availability

All data generated or analyzed during this study are included in this published article.

## Abbreviations

3-MA: 3-Methyladenine
AAV9: Recombinant adeno-associated virus serotype 9
DCM: Diabetic cardiomyopathy
Eco: Echocardiography
EF: Ejection fraction
ER: Endoplasmic reticulum
FS: Fractional shortening
GRP78: Glucose-regulated protein 78
GSK: GSK2656157
HG: High glucose
Mel: Melatonin
PERK: Protein kinase RNA -like ER kinase
T1DM: Type 1 diabetes mellitus
UPR: Unfolded protein response
VEGF-B: Vascular endothelial growth factor-B
