# Supplementary material for: Melatonin attenuates diabetic cardiomyopathy by increasing autophagy of cardiomyocytes via regulation of VEGF-B/GRP78/PERK signaling pathway
Source: Cardiovasc Diabetol. 2024 Jan 9;23:19. doi: 10.1186/s12933-023-02078-x (PMC10777497; doi:10.1186/s12933-023-02078-x)
Supplement: Supplementary file 1 — Additional file 1: Figure S1. The metabolic parameters for WT mice. (A) The experimental protocol in WT mice. (B-C) Fasting and non-fasting blood glucose levels, n=8. (D) IPGTT was performed at week 8, n=6. (E) Area of under curve (AUC) of IPGTT was calculated, n=6. (F-H) Body weight, food intake and water intake were obtained, n=8. Data were expressed as the mean ± SD. ***p<0.001, ****p<0.0001. One-way ANOVA followed by a post hoc Tukey’s test. Figure S2. Construction of global VEGF-B knock out mice and AAV-VEGF-B overexpression mice. (A) PCR analysis of VEGF-B genotype for the presence of WT, heterozygotes (vegf-b+/-) and homozygous (vegf-b-/-). (B) mRNA levels of Vegfb in WT, VEGFB+/- and VEGF-B-/- mice, n=5. (C) mRNA levels of Vegfb in WT, Vector and AAV-VEGF-B mice, n=5. (D) We detected Flag-tag in AAV injection mice heart by western blot. (E) The immunohistochemical analysis for VEGF-B in mice myocardial tissues (scale bar=100μm). (F) The experimental protocol for WT VEGF-B-/- and AAV-VEGF-B mice. (G) mRNA levels of Vegfb in mice, n=5. Data were expressed as the mean ± SD. ****p<0.0001. One-way ANOVA followed by a post hoc Tukey’s test. Figure S3. The metabolic parameters for WT, VEGF-B-/- and AAV-VEGF-B mice. (A, B) fasting and none-fasting blood glucose, n=8. (C) IPGTT was performed at week 8, n=6. (D) AUC of IPGTT was calculated, n=6. (E-F) Body weight, food intake and water intake were obtained, n=8. Data were expressed as the mean ± SD. ***p<0.001, ****p<0.0001. One-way ANOVA followed by a post hoc Tukey’s test. Figure S4. The absence of VEGF-B or AAV-VEGF-B did not affect the autophagy in normal mice. (A) Western blot for VEGF-B, p62 and LC3 in mice. Quantification for p62, VEGF-B and LC3 II/I in mice, n=3. (B) Western blot for VEGF-B, p62 and LC3 in NRVMs. Quantification for p62, VEGF-B and LC3 II/I in NRVMs, n=3. (C) Western blot for VEGF-B, p62 and LC3 II/I in VEGF-B-/- and AAV-VEGF-B mice in mice heart by western blot, n=3. (D) Quantification for p62 [file 12933_2023_2078_MOESM1_ESM.docx]

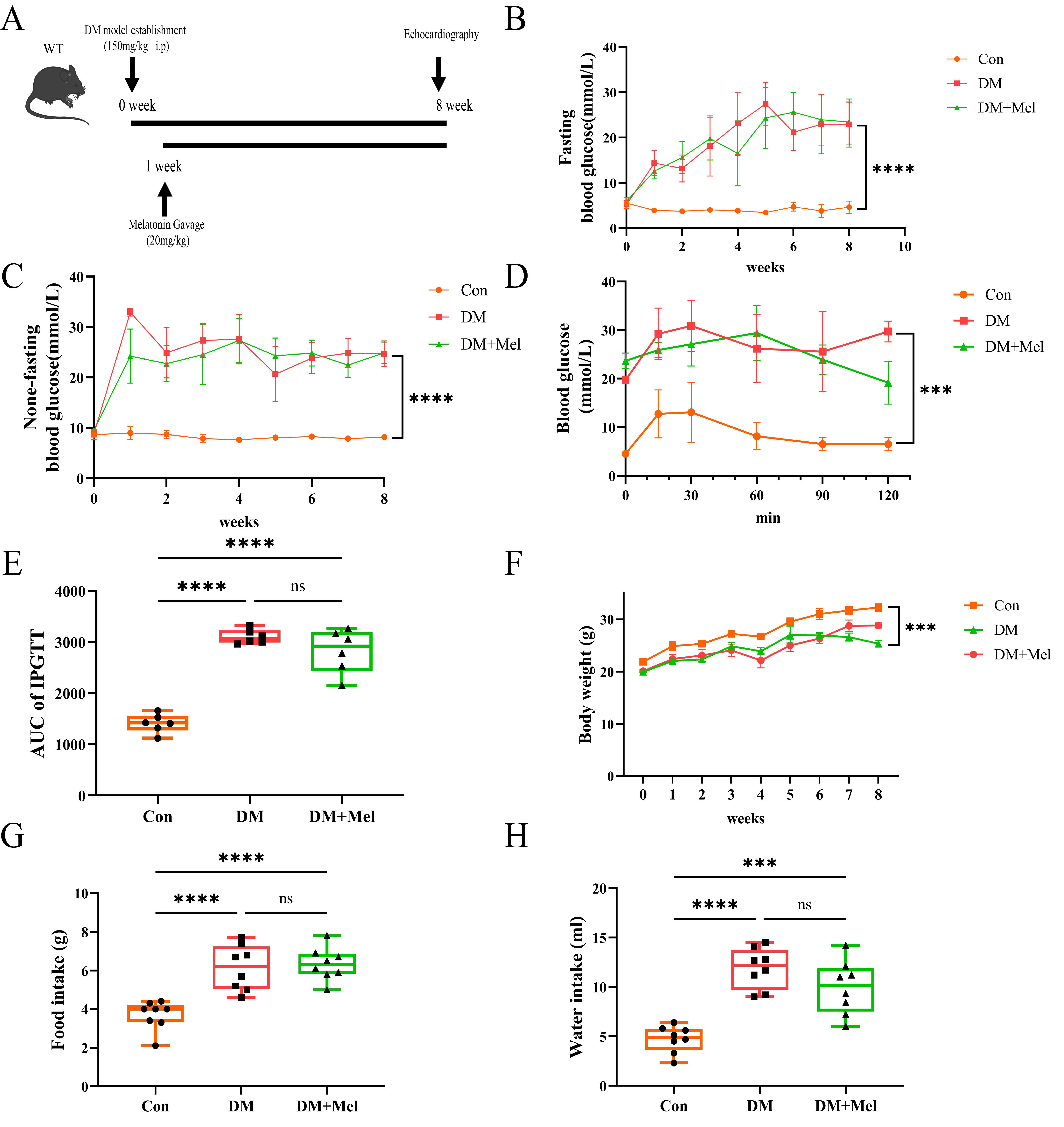


**Figure S1. The metabolic parameters for WT mice.** (A) The experimental protocol in WT mice. (B-C) Fasting and non-fasting blood glucose levels, n=8. (D) IPGTT was performed at week 8, n=6. (E) Area of under curve (AUC) of IPGTT was calculated, n=6. (F-H) Body weight, food intake and water intake were obtained, n=8. Data were expressed as the mean ± SD. ***p<0.001, ****p<0.0001. One-way ANOVA followed by a post hoc Tukey’s test.


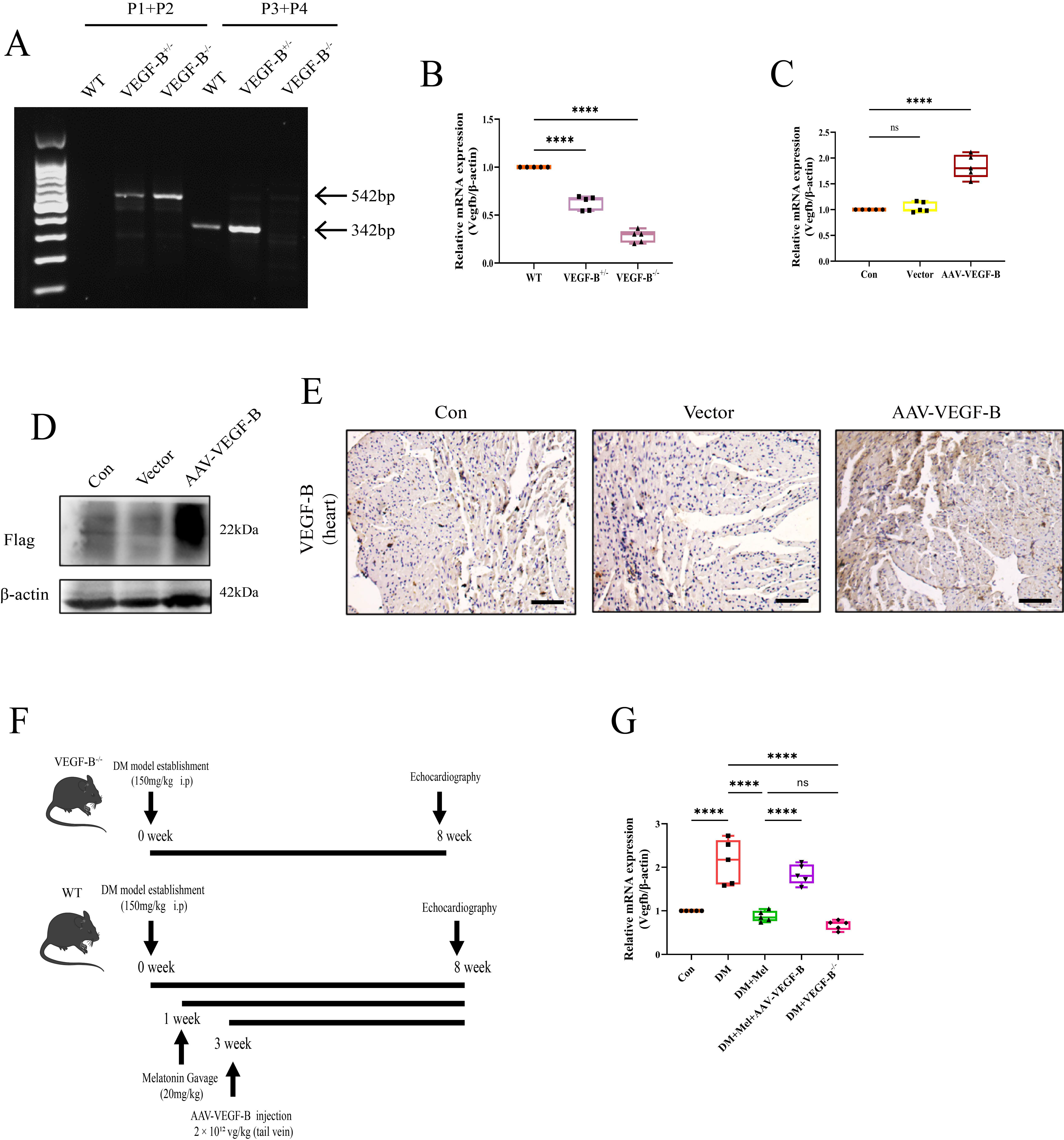


**Figure S2. Construction of global VEGF-B knock out mice and AAV-VEGF-B overexpression mice.** (A) PCR analysis of VEGF-B genotype for the presence of WT, heterozygotes (vegf-b^+/-^) and homozygous (vegf-b^-/-^). (B) mRNA levels of *Vegfb* in WT, VEGFB^+/-^ and VEGF-B^-/-^ mice, n=5. (C) mRNA levels of *Vegfb* in WT, Vector and AAV-VEGF-B mice, n=5. (D) We detected Flag-tag in AAV injection mice heart by western blot. (E) The immunohistochemical analysis for VEGF-B in mice myocardial tissues (scale bar=100μm). (F) The experimental protocol for WT VEGF-B^-/-^ and AAV-VEGF-B mice. (G) mRNA levels of *Vegfb* in mice, n=5. Data were expressed as the mean ± SD. ****p<0.0001. One-way ANOVA followed by a post hoc Tukey’s test.


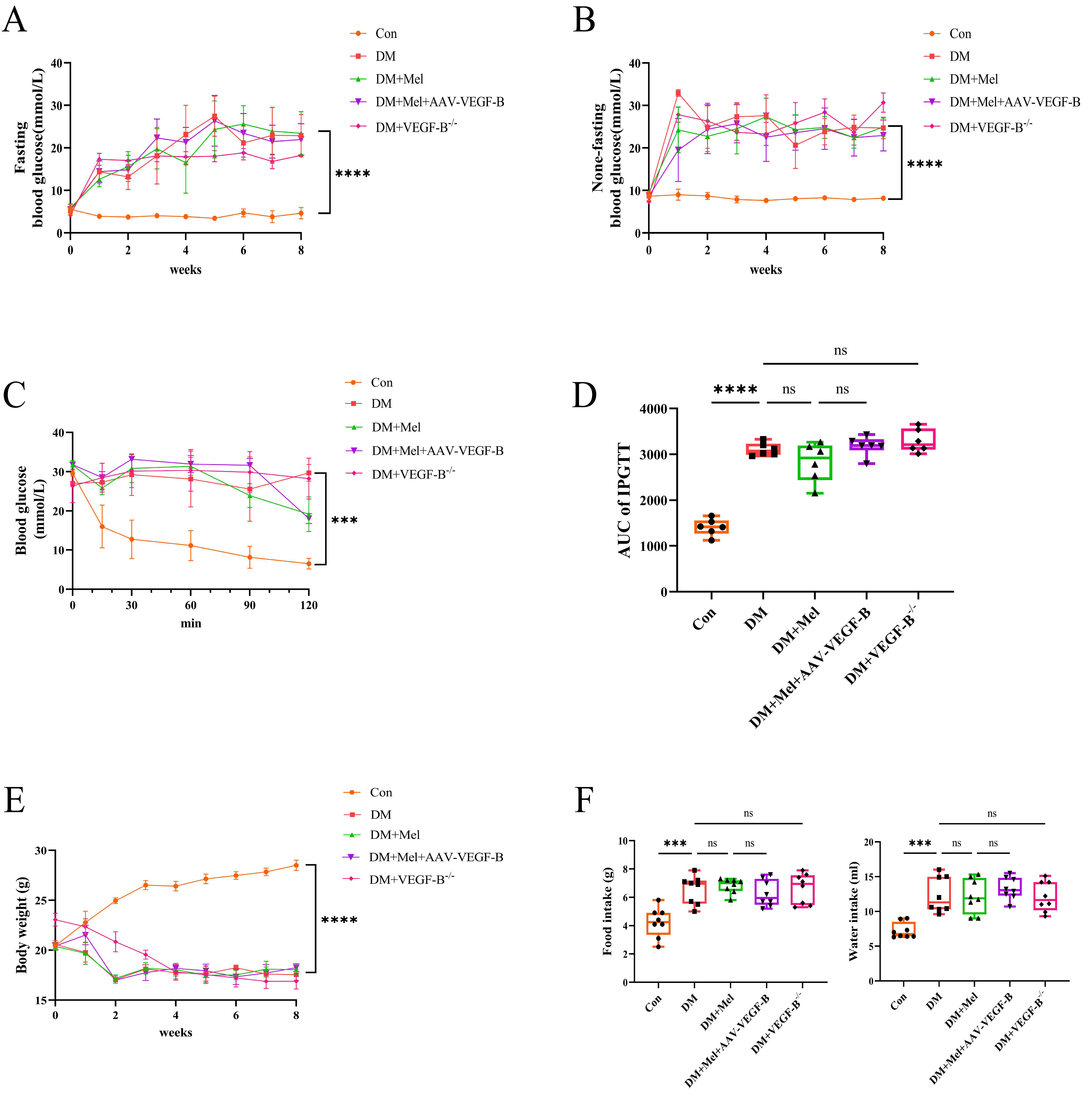


**Figure S3. The metabolic parameters for WT, VEGF-B^-/-^ and AAV-VEGF-B mice.** (A, B) fasting and none-fasting blood glucose, n=8. (C) IPGTT was performed at week 8, n=6. (D) AUC of IPGTT was calculated, n=6. (E-F) Body weight, food intake and water intake were obtained, n=8. Data were expressed as the mean ± SD. ***p<0.001, ****p<0.0001. One-way ANOVA followed by a post hoc Tukey’s test.


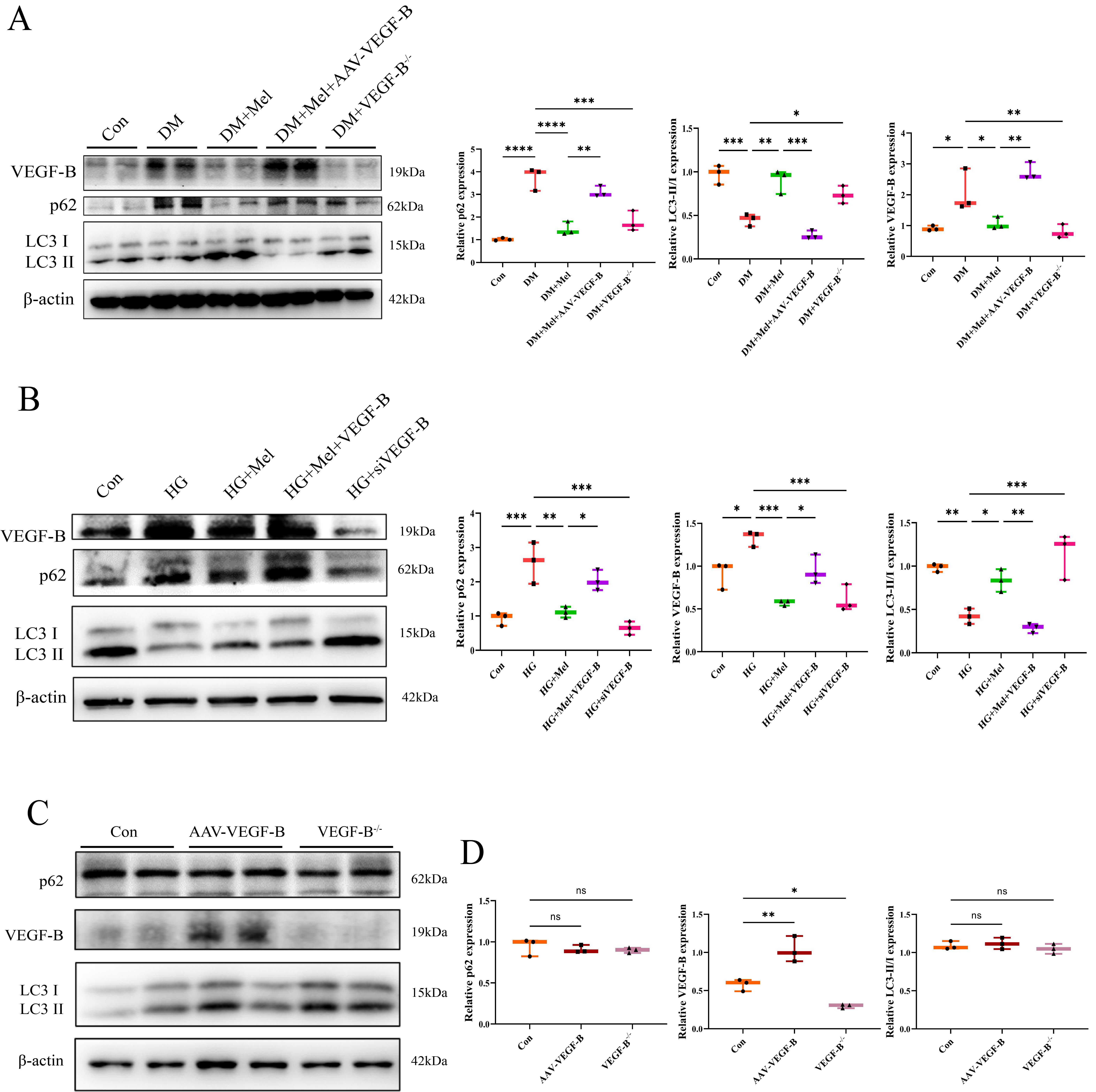


**Figure S4. The absence of VEGF-B or AAV-VEGF-B did not affect the autophagy in normal mice.**

(A) Western blot for VEGF-B, p62 and LC3 in mice. Quantification for p62, VEGF-B and LC3 II/I in mice, n=3. (B) Western blot for VEGF-B, p62 and LC3 in NRVMs. Quantification for p62, VEGF-B and LC3 II/I in NRVMs, n=3. (C) Western blot for VEGF-B, p62 and LC3 II/I in VEGF-B^-/-^ and AAV-VEGF-B mice in mice heart by western blot, n=3. (D) Quantification for p62, VEGF-B and LC3 II/I, n=3. Data were expressed as the mean ± SD. *p<0.05, **p<0.01, ***p<0.01, ****p<0.0001. One-way ANOVA followed by a post hoc Tukey’s test.


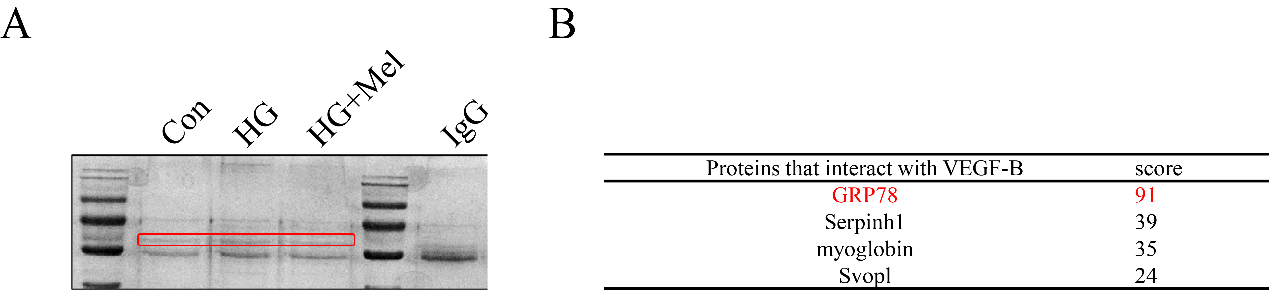


**Figure S5. IP-MS in NRVMs with high glucose treatment to find VEGF-B interactors.** (A) Coomassie brilliant blue staining. (B) The candidates for VEGF-B interactors.


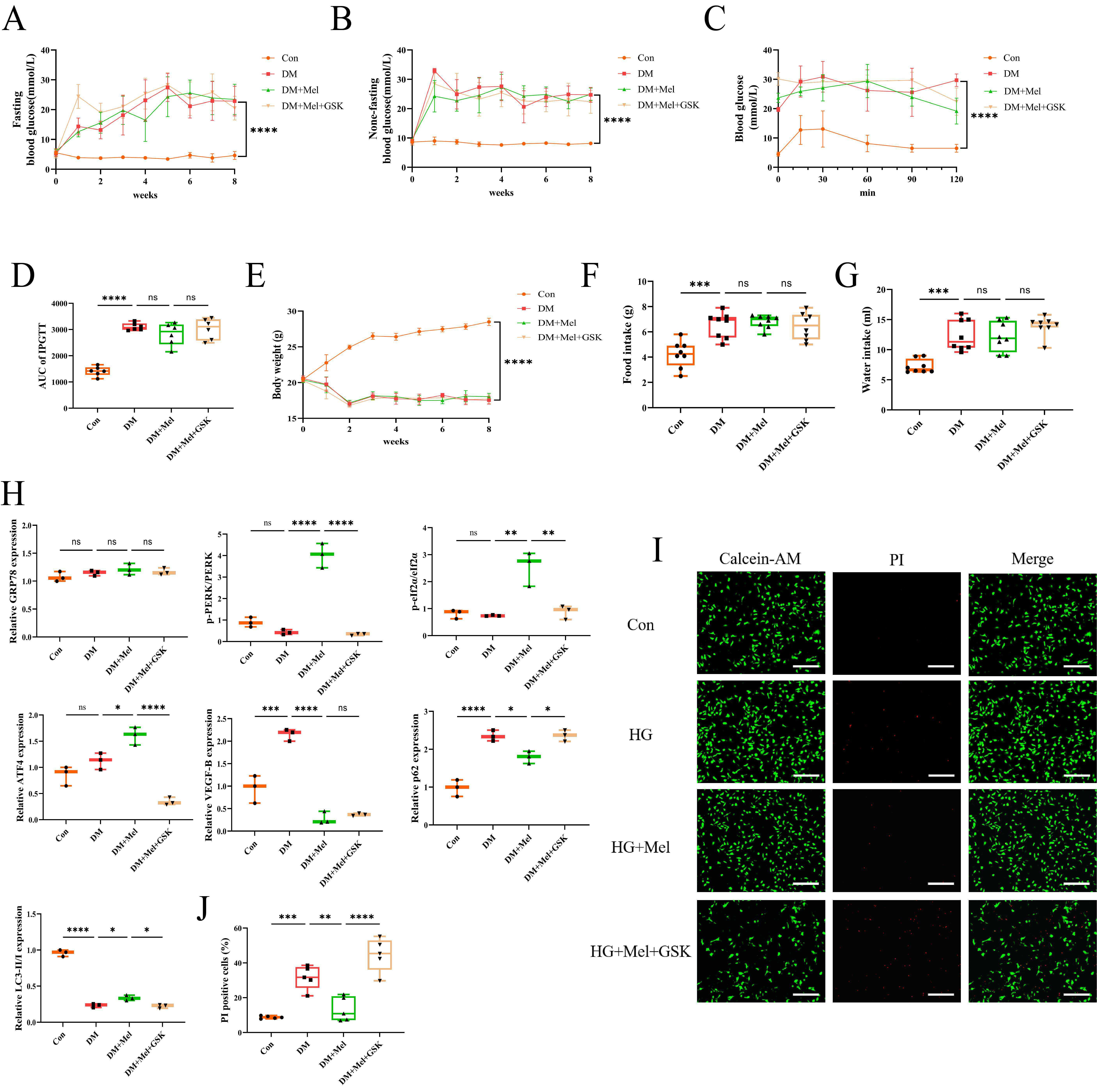


**Figure S6. The metabolic parameters for GSK treated mice.** (A, B) Fasting and none-fasting blood glucose, n=8. (C) IPGTT was performed at week 8, n=6. (D) AUC of IPGTT was calculated, n=6. (E-G) Body weight, food intake and water intake were obtained, n=8. (H) Quantification for PERK-related pathways and autophagy proteins in mice, n=3. (I) Calcein-AM/PI double staining in NRVMs (scale bar=200μm). (J) Quantification of PI-positive cells, n=5. Data were expressed as the mean ± SD. *p<0.05, **p<0.01, ***p<0.001, ****p<0.0001. One-way ANOVA followed by a post hoc Tukey’s test.


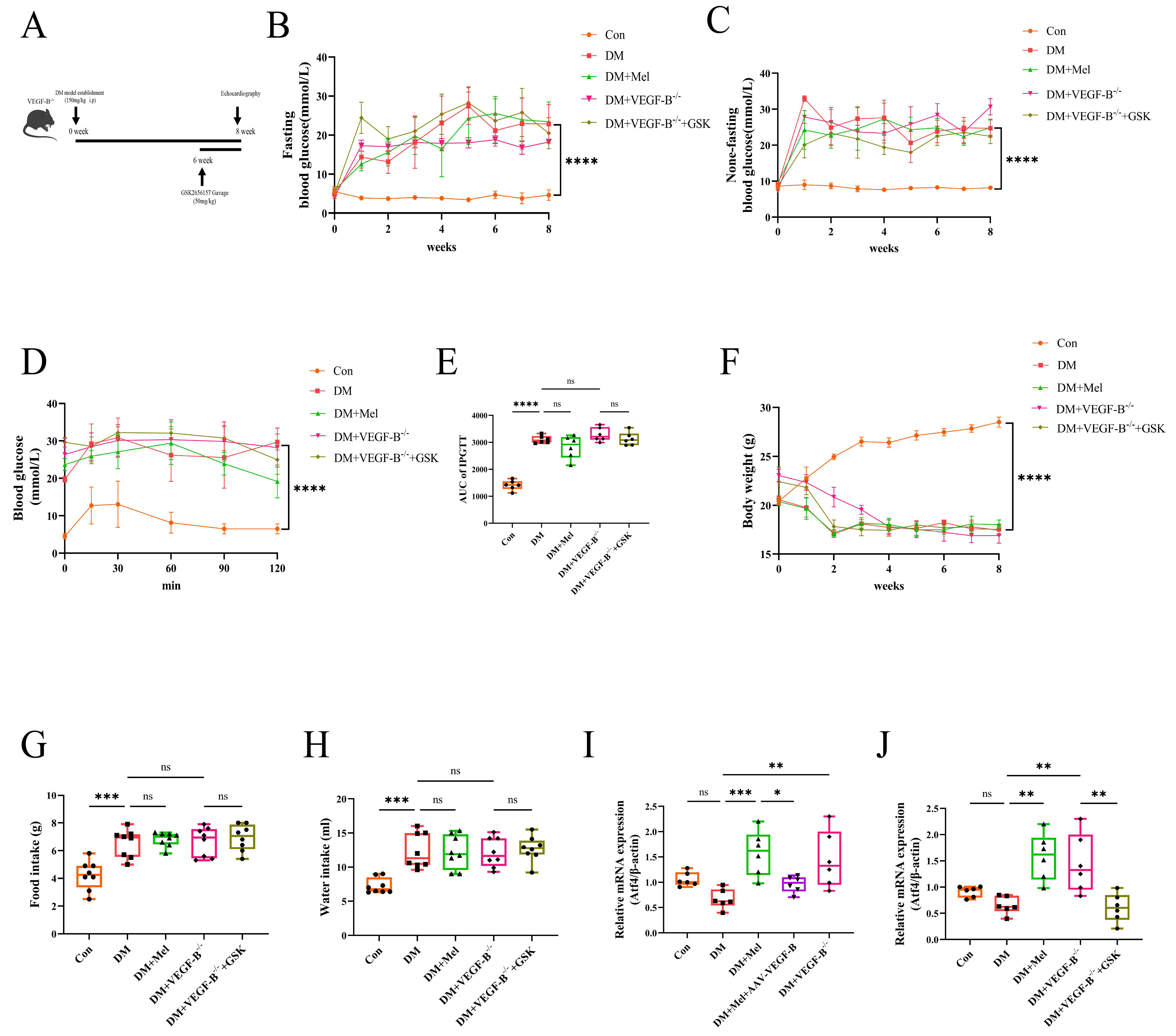


**Figure S7. The metabolic parameters in mice.** (A) The experimental protocol for VEGF-B^-/-^ and GSK treatment mice. (B, C) Fasting and none-fasting blood glucose, n=8. (D) IPGTT was performed at week 8, n=6. (E) AUC of IPGTT was calculated, n=6. (F-H) Body weight, food intake and water intake were obtained, n=8. (I, J) mRNA levels of *Atf4* in AAV-VEGF-B, VEGF-B^-/-^ and GSK treatment mice, n=6. Data were expressed as the mean ± SD. *p<0.05, **p<0.01, ***p<0.001, ****p<0.0001. One-way ANOVA followed by a post hoc Tukey’s test.
