# Supplementary material for: Melatonin attenuates diabetic cardiomyopathy by increasing autophagy of cardiomyocytes via regulation of VEGF-B/GRP78/PERK signaling pathway
Source: Cardiovasc Diabetol. 2024 Jan 9;23:19. doi: 10.1186/s12933-023-02078-x (PMC10777497; doi:10.1186/s12933-023-02078-x)
Supplement: Supplementary file 2 — Additional file 2: Table S1. Genotyping of WT, VEGF-B+/- and VEGF-B-/-. [file 12933_2023_2078_MOESM2_ESM.docx]

**Table S1 Genotyping of WT, VEGF-B^+/-^ and VEGF-B^-/-^**

| Genotype  Primer | WT | VEGF-B^+/-^ | VEGF-B^-/-^ |
| --- | --- | --- | --- |
| P1+P2 (542bp) | **-** | **+** | **+** |
| P3+P4 (342bp) | **+** | **+** | **-** |
