# Supplementary material for: Melatonin attenuates diabetic cardiomyopathy by increasing autophagy of cardiomyocytes via regulation of VEGF-B/GRP78/PERK signaling pathway
Source: Cardiovasc Diabetol. 2024 Jan 9;23:19. doi: 10.1186/s12933-023-02078-x (PMC10777497; doi:10.1186/s12933-023-02078-x)
Supplement: Supplementary file 3 — Additional file 3: Table S2. Echocardiographic parameters in mice. [file 12933_2023_2078_MOESM3_ESM.docx]

Table S2 Echocardiographic parameters in mice

| Groups | Con | DM | DM+Mel | DM+Mel+AAV-VEGF-B | DM+VEGF-B^-/-^ | DM+Mel+GSK | DM+VEGF-B^-/-^+GSK |
| --- | --- | --- | --- | --- | --- | --- | --- |
| Number of animals | n=6 | n=6 | n=6 | n=6 | n=6 | n=6 | n=6 |
| Heart rate, (bmp) | 452±43 | 471±41 | 462±55 | 451±35 | 460±32 | 418±27 | 474±39 |
| Diameter;s, (mm) | 1.2±0.1 | 2.0±0.3 | 1.8±0.3 | 2.7±0.3 | 1.4±0.3 | 2.5±0.2 | 2.4±0.4 |
| Diameter;d, (mm) | 2.7±0.2 | 3.2±0.3 | 3.2±0.4 | 3.5±0.2 | 3.3±0.4 | 3.1±0.3 | 3.3±0.2 |
